# Activation of embryonic/germ cell-like axis links poor outcomes of gliomas

**DOI:** 10.1186/s12935-022-02792-8

**Published:** 2022-11-26

**Authors:** Zhan Ma, Fengyu Zhang, Ji Xiong, Haishi Zhang, Hui-Kuan Lin, Chunfang Liu

**Affiliations:** 1 Department of Laboratory Medicine, Shanghai Medical College, Huashan Hospital, Fudan University, Shanghai, 200040 China; 2 Department of Laboratory Medicine, Shanghai Children’s Hospital, Shanghai Jiao Tong University, Shanghai, 200040 China; 3 Department of Pathology, Shanghai Medical College, Fudan University, Shanghai, 200040 China; 4 Department of Neurosurgery, Huashan Hospital, Fudan University, Shanghai, 200040 China; 5 Department of Cancer Biology, Wake Forest School of Medicine, Winston-Salem, NC 27157 USA

**Keywords:** Gametogenesis hypothesis of tumours, Glioma prognosis, Embryonic/germ cell-like cycle, Primordial germ cell-like tumor cells, Markers for poor outcomes of glioma patients

## Abstract

**Background:**

It is unclear which core events drive the malignant progression of gliomas. Earlier studies have revealed that the embryonic stem (ES) cell/early PGC state is associated with tumourigenicity. This study was designed to investigate the role of ES/PGC state in poor outcomes of gliomas.

**Methods:**

Crispr-Cas9 technology, RT–PCR and animal experiments were used to investigate whether PGC-like cell formation play crucial roles in the tumorigenicity of human glioma cells. Bioinformatic analysis was used to address the link between ES/PGC developmental axis and glioma overall outcomes.

**Results:**

Here, our findings showed that germ cell-like cells were present in human gliomas and cultured glioma cells and that the formation of germ cell-like cells was essential for glioma tumours. Bioinformatic analysis showed that the mRNA levels of genes related to embryonic/germ cell development could be detected in most gliomas. Our findings showed that the activation of genes related to reprogramming or the germ cell-like state alone seemed to be insufficient to lead to a malignant prognosis, whereas increased mRNA levels of genes related to the activation of the embryonic/germ cell-like cycle (somatic PGC-EGC-like cycle and somatic parthenogenetic embryo-like cycle) were positively correlated with malignant prognoses and poor clinical outcomes of gliomas. Genes related to the embryonic/germ cell cycle alone or in combination with the WHO grade or 1p19q codeletion status could be used to subdivide gliomas with distinct clinical behaviours.

**Conclusion:**

Together, our findings indicated that a crucial role of germ cell-like cell formation in glioma initiation as well as activation of genes related with the parthenogenetic embryo-like cycle and PGC-EGC-like cycle link to the malignant prognosis and poor outcomes of gliomas, which might provide a novel way to better understand the nature of and develop targeted therapies for gliomas as well as important markers for predicting clinical outcomes in gliomas.

**Supplementary Information:**

The online version contains supplementary material available at 10.1186/s12935-022-02792-8.

## Introduction

Despite differences in their tissues of origin and genetic backgrounds, many tumours exhibit common phenotypes, such as high embryonic/germ cell traits, which is accounted for in the embryo/gametogenesis-related hypothesis of tumours proposed by Müller (1838), Langenbeck (1840) and Beard (1902) and extended by Old (2001) [1–5]. This hypothesis postulated that tumours arise from germ cells or reactivation of the germ cell programme in somatic tissues. Earlier studies have revealed that tumourigenicity is an inherent feature of the embryonic stem (ES) cell/early primordial germ cell (PGC) state, including early PGCs, implantation embryos, parthenogenetic oocytes, embryonal carcinoma (EC) cells, embryonic stem (ES) cells, embryonic germ cells (EGCs) and induced pluripotent stem (iPS) cells [6–11], raising the possibility that re-obtaining the ES/early PGC state may be one of the driving events in the malignant behaviours of somatic tumours.

**Fig. 1:**
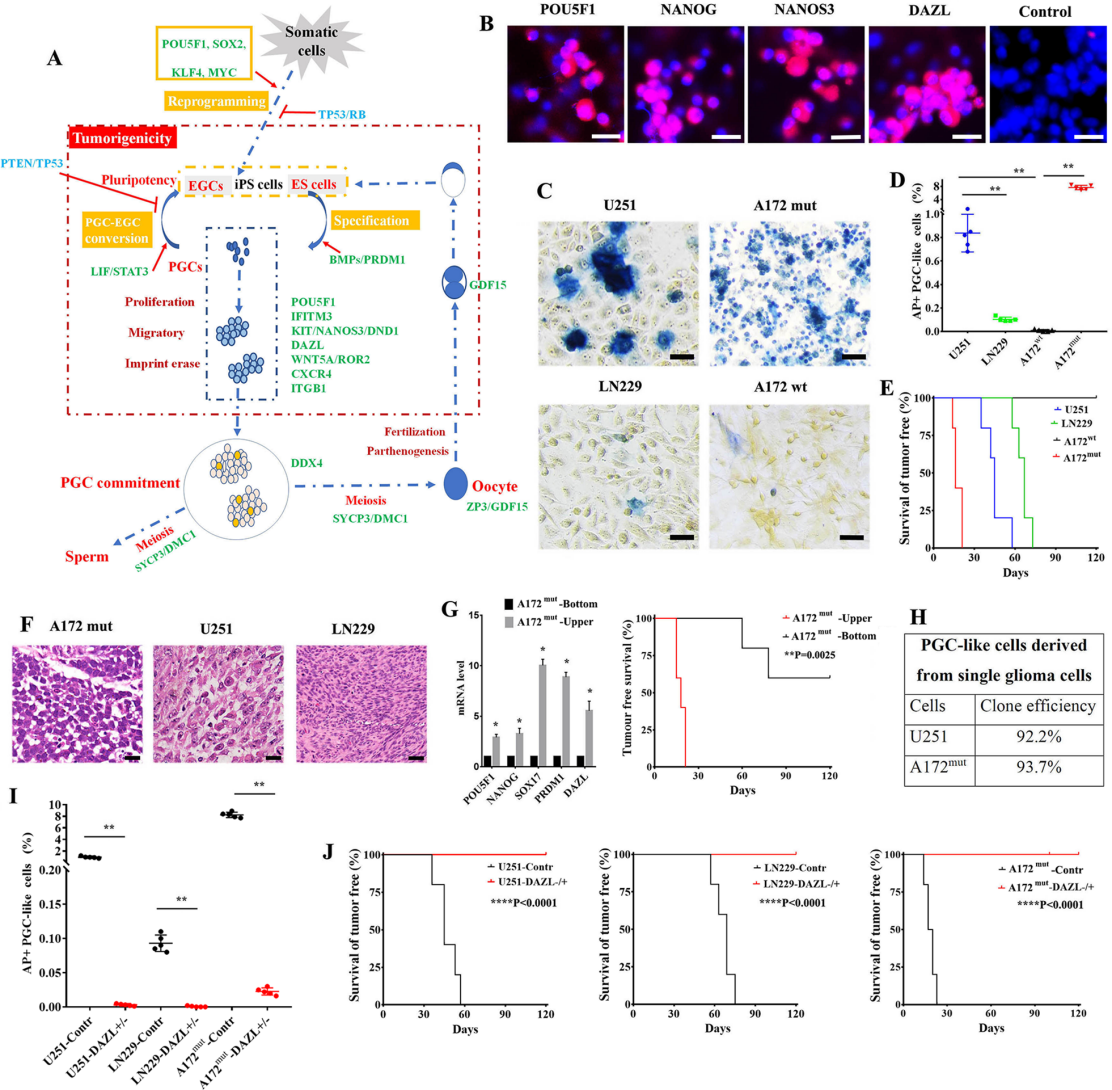
Experimental hypothesis and the crucial role of germ cell-like cell formation in tumour initiation. **A** Experimental hypothesis. **B** Immunofluorescence assays showed the expression and colocalization of the indicated antigens in cultured U251 cells. **C** AP staining showing PGC-like cells in U251, LN229, A172^wt^ and A172^mut^ glioma cells. **D** The percentage of AP-positive cells in glioma cultured cells. **E** Survival curve of tumour-free glioma cells showing their different tumour initiation abilities. **F** Image of tumour tissues (HE staining) from grafted mice. **G** The comparison of indicated gene expression and tumour initiating ability between A172 bottom cells and A172 upper cells. (**H**) The efficiency of generating clones of PGC-like cells from single glioma cells. **I** The percentage of AP-positive cells in the control and *DAZL*^+/−^ glioma cells. **J** The Survival curve of tumour-free cells showing the tumour initiation ability of the control and *DAZL*^+/−^ glioma cells. None of the *DAZL*^+/−^ cell lines could cause tumours within 100 days after grafting in nude mice. Scale bar = 20 μm in (**F**), 50 μm in (**B**, **C**). **P < 0.01

An increasing number of studies have revealed that embryonic/germ cell-specific genes play crucial roles in tumourigenicity, metastasis and therapy resistance [12–25]. Notably, it has been shown that knockout of those genes related to germ cell development fully inhibits brain tumour formation in *Drosophila* [26], and no melanoma initiation occurs before the reactivation of genes related to embryonic development in a zebrafish model [27]. Our previous studies showed that embryonic/germ cell-like tumour cells were found in various types of tumours, played important roles in tumour growth, liver metastasis and drug resistance; and exhibited an independent life cycle [19–21, 28–31]. We also showed that the formation of embryonic/germ cell-like tumour cells was induced by either p53 deficiency or a chemical carcinogen (3-methylcholanthrene, 3-MCA) [20, 28]. In essence, PGCs give rise to sperm or oocytes and then return to the embryonic state via fertilization or parthenogenesis. However, PGCs can also return to the embryonic state via PGC-EGC conversion under some conditions, such as *PTEN/TP53/SOX17* deficiency [9, 32, 33]. Therefore, we postulated that the acquisition and maintenance of the ES/early PGC state via activation of the embryonic/germ cell-like developmental axis might be linked to the core malignant behaviours of somatic tumours through somatic cell-ESC/PGC-(post-migratory PGC)-EGC-somatic cell-like conversion (which we named the somatic PGC-EGC/ES-like cycle) and/or somatic tumour cell-ESC/PGC-(post-migratory PGC)-oocyte-parthenogenetic embryo-somatic tumour cell conversion (which we named the somatic parthenogenetic embryo-like cycle) [20] (Fig. 1A). Here, our findings showed that activation of the embryonic/germ cell-like development axis was essential in the malignant progression of gliomas and that related genes could be used as important markers to predict clinical outcomes in gliomas.

## Methods

### Cell culture

U251, LN229 and A172^wt^ cells were obtained from ATCC. A172^mut^ cells were derived from A172^wt^ cells after treatment with a chemical carcinogen (3-methylcholanthrene, 3-MCA). All cells were cultured in high-glucose Dulbecco’s modified Eagle’s medium (DMEM, HyClone) with 10% foetal bovine serum (FBS; Sigma) and 1% L-glutamine and were maintained at 37 °C with 5% CO_2_.

### Single cell cloning

U251 or A172^mut^ cells were plated in 96-well plates by the limited dilution method and incubated at 37 °C with 5% CO_2_ for proliferation. Wells with single cells were selected and then treated with AP staining after culturing for 3 weeks. The efficiency of generating PGC-like cells was counted in the single clones.

### Alkaline phosphatase staining

Cultured cells were fixed with 4% paraformaldehyde in PBS for 4 min, washed twice with a Tris-HCl (pH = 8.6) buffer solution and then incubated with AP substrate (Vector laboratory) for 40 min at room temperature.

### Real-time PCR analysis

RNA was isolated from cultured cells by Trizol reagent (Invitrogen) and then converted to cDNA by reverse transcription using a reverse transcription kit (Invitrogen). Real-time PCR analysis was performed with a SYBR Green PCR Master Mix Kit (Applied Biosystems) according to the manufacturer’s instructions.

### Animal experiments

All animal protocols were carried out in accordance with ARRIVE guidelines.

To compare tumorigenicity, the U251, LN229, A172^wt^ and A172^mut^ cells, U251-*DAZL*^+/−^, LN229-*DAZL*^+/−^, A172mut-*DAZL*^+/−^ cells, U251-guide, LN229-guide and A172^mut^-guide (1.5 × 10^5^ cells/mice) were subcutaneously injected into female nude mice (n = 5, age 6 weeks, body weight 20–22 g) respectively. The time of tumour initiation was recorded every day. Five mice were raised in a cage under specific pathogen-free conditions and a 12-h light/dark cycle at 23 ± 2 °C and 60 ± 10% humidity, and standard food and water were freely available. At the end of the experiment, the mice were euthanized via exposure to a gradually increasing concentration of carbon dioxide gas in accordance to ARRIVE guidelines and then resected the subcutaneous tumors which were then fixed in 4% formaldehyde for pathological analysis. A P-value < 0.05 was considered to be significant.

### Immunochemistry

Cells were cultured on coverslips. fixed with 4% paraformaldehyde and then blocked in PBS with 5% BSA and 0.05% Triton-X-100. Cells or tissue sections were incubated overnight at 4 °C with various primary antibodies, including anti-POU5F1 (ab184665, Abcam), anti-Sox2 (MAB2018R-100, R&D), anti-Nanog (NB100-58842, Novus Biologicals), anti-Nanos3 (ab70001, Abcam), anti-DDX4 (ab27591, Abcam), SCP3 (ab 15,093, Abcam) or anti-*DAZL* (NB100-2437, Novus biologicals). Next, the cells were stained with Cy3 fluorescent dye-conjugated secondary antibodies (Jackson) and 4,6-diamidino-2-phenylindole (DAPI; Invitrogen), while the tissue sections were stained with haematoxylin. The cells just were stained with Cy3 fluorescent dye-conjugated secondary antibodies (Jackson) and 4,6-diamidino-2-phenylindole (DAPI; Invitrogen) as control.

### *DAZL* deletion

The CRISPR/Cas9 and sgRNA-RFP plasmids were provided by Addgene and Sigma, respectively [24]. The *DAZL*-sgRNAs sequences are: GAAGCTTCTTTGCTAGATATGG (Additional file 1: Table S1). The guide empty vector was used as the blank control (Sigma). To delete *DAZL* in U251, LN229 and A172^mut^ cells, we transfected glioma cells simultaneously with the CRISPR/Cas9 vector and guide RNAs (gRNAs) as described previously [21, 24]. Guide gene was used as a knockout control. Two days post transfection, the treated glioma cells were selected with 4 µg/ml of blasticidin or 5 µg/ml of puromycin for 5 days. Single cells were then isolated from the selected cells by dilution and clonally expanded. Single clone cells with *DAZL* deletion were selected by gene sequencing. The clone cells with *DAZL* frameshift mutation were *DAZL*^+/−^ (Additional file 1: Fig. S1). We got 9 *DAZL*^+/−^ U251 cells, 3 *DAZL*^+/−^ LN229 cells and 7 *DAZL*^+/−^ A172^mut^ cells.

### Tissue specimens

Glioma tissues were obtained from 38 patients with gliomas, including 20 grade IV gliomas, 8 grade III gliomas and 10 grade II gliomas. All patients underwent curative resection at Huashan Hospital of Fudan University from 2011 to 2013. Glioma tissues were stained with hematoxylin eosin (HE) or antibody.

### Data of the CGGA database

Data from the Cancer Genome Atlas (TCGA), the Genotype-Tissue Expression (GTEx) and the Chinese Glioma Genome Atlas (CGGA) (http://cgga.org.cn/index.jsp) were used to confirm our concept. The Data from the Cancer Genome Atlas (TCGA) and the Genotype-Tissue Expression (GTEx) were analysed using the http://gepia2.cancer-pku.cn/#analysis. The WEseq_286 dataset was used to analyse the genetic mutations. The methyl_159 dataset was used to analyse the methylation status of various genes. The mRNAseq_325 and mRNAseq_693 datasets (Additional file 1: Table S2) were used to analyse the mRNA levels of various genes as well as to investigate whether the mRNA level was associated with the pathologic grades or outcomes of gliomas. The mRNA-array_301 dataset was used to analyse the mRNA level of *DAZL* in gliomas and its link to the pathologic grades and outcomes of gliomas. Data without overall survival information were deleted. Detailed information on the datasets for mRNA sequencing is provided in Additional file 1: Table S2.

### Statistical analysis

Kaplan–Meier curves were used to assess overall survival for each of the genes and test groups. Unpaired t test with Welch’s correction and Mann Whitney test were used to analyse the difference of gene expression between distinct groups.

## Results

### A crucial role of germ cell-like cell formation in tumour initiation

PGC-like cells were frequently observed in U251 and A172 mutant (A172 ^mut^) glioma cell cultures but were barely observed in L229 and A172 wild-type (A172 wt) glioma cell cultures (Fig. 1B–D, Additional file 1: Fig. S2A, B). We found that germ cell-like cell formation was positively correlated with tumour initiation in glioma cells, including U251, LN229, A172 ^wt^ and A172 ^mut^ cells (Fig. 1D and E). Notably, A172 ^wt^ cells without germ cell-like cell properties failed to form tumour within 120 days after grafting in nude mice. However, A172 ^mut^ cells that reobtained germ cell-like properties after treatment with a chemical carcinogen (3-methylcholanthrene, 3-MCA) gave rise to tumours within 2 weeks of grafting in nude mice (Fig. 1E). Interestingly, tumour tissues derived from A172 ^mut^ cells were teratocarcinomas (Fig. 1F), which provided strong support for the presence of germ cell-like cells since teratocarcinomas are thought to originate from germ cells [2, 5]. Germ cell-like tumour cells often grow above somatic tumour cells and can be easily separated from somatic tumour cells in vitro. We separated the upper cultures, which were enriched with PGC-like cells, from the bottom cultures, which contained a small proportion of PGC-like cells in A172^mut^ cells, by shaking the flask rigorously. RT–PCR data showed that the expression of genes related to germ cells was much higher in the upper cells than in the bottom cells in A172 ^mut^ cultures (Fig. 1G). Compared to the bottom cells, the upper cells in A172 ^mut^ cultures showed increased tumour initiation abilities after injection into nude mice (Fig. 1G). Notably, SOX17 which links to the PGC state of human were reactivated in the upper cells of A172 ^mut^ cultures and expressed highly versus SOX2 which links to ES state of human (Additional file 1: Fig. S2C). After culture, most single U251 and A172 ^mut^ cells generated clones containing PGC-like cells, indicating that PGC-like cells could be derived from somatic glioma cells (Fig. 1H).

We then investigated the relationship between germ cell formation and tumour initiation by deleting *DAZL*, which plays a crucial role in germ cell development from PGC specification, PGC fate determination to PGC further mature [34–36], with CRISPR-Cas9 technology. Since *DAZL*^−/−^ glioma cells were not viable in culture possibly attributed to a crucial role of DAZL or germ cell fate in immortality of tumour cells, U251- *DAZL*^+/−^, LN229-*DAZL*^+/−^ and A172^mut^- *DAZL* ^+/−^ cells were studied (Additional file 1: Table S2). Our earlier study revealed that knockdown of *DAZL* inhibited tumour formation and increased the therapeutic sensitivity of cultured human glioma cells [24]. In this study, we further revealed that knockdown of *DAZL* greatly impaired germ cell formation and tumour initiation in U251, LN229 and A172 ^mut^ cells (Fig. 1I and J). These findings showed that activation of PGC-like cell formation from somatic tumour cells was essential in the initiation of glioma cell tumours, leading to tumourigenicity in new sites. Thus, activation of the PGC-like state might be necessary for a more aggressive stage of gliomas.

### Genetic and epigenetic changes in genes related to embryonic/germ cell development in gliomas

PGC specification arises from ES cells [36–38], and findings in iPS cells highlight the possibility of somatic cell reprogramming [11]. This means that if a PGC-like fate truly occurs in gliomas, at least two key events are possibly involved: reprogramming and PGC specification (Fig. 1A). Therefore, we investigated whether the genes related to reprogramming, PGC specification and PGC development (including conversion of PGCs into EGCs and oogenesis from PGCs) were activated in gliomas and linked to the malignant prognosis of gliomas (Fig. 1A). The defined gene groups included those involved in reprogramming (inhibition: TP53; promotion: *POU5F1, SOX2, MYC, KLF4*), pluripotency (*POU5F1, SOX2, MYC, KLF4, NOTCH1*), induction of ES-PGC conversion by microenvironment (*BMP2, BMP4, BMP8B, LIF*), PGC specification (*POUF51, PRDM1, SOX17, ACVR1, IFITM3*), PGC fate maintenance (*SOX17, PRDM1, KIT, Nanos3, DND1*), PGC survival (*DAZL, DDX4*), proliferation/migration/survival (*ITGB1, CXCR4, WNT5A, ROR2*), meiosis (*SYCP3, DMC1*), PGC-EGC conversion (inhibition: *TP53, PTEN, BMP2, BMP4, SOX17*; promotion: *LIF, STAT3*), oocytes (*ZP3, GDF15*) as well as early embryos (*GDF15*) [11, 33, 36, 37, 39–44].

**Fig. 2:**
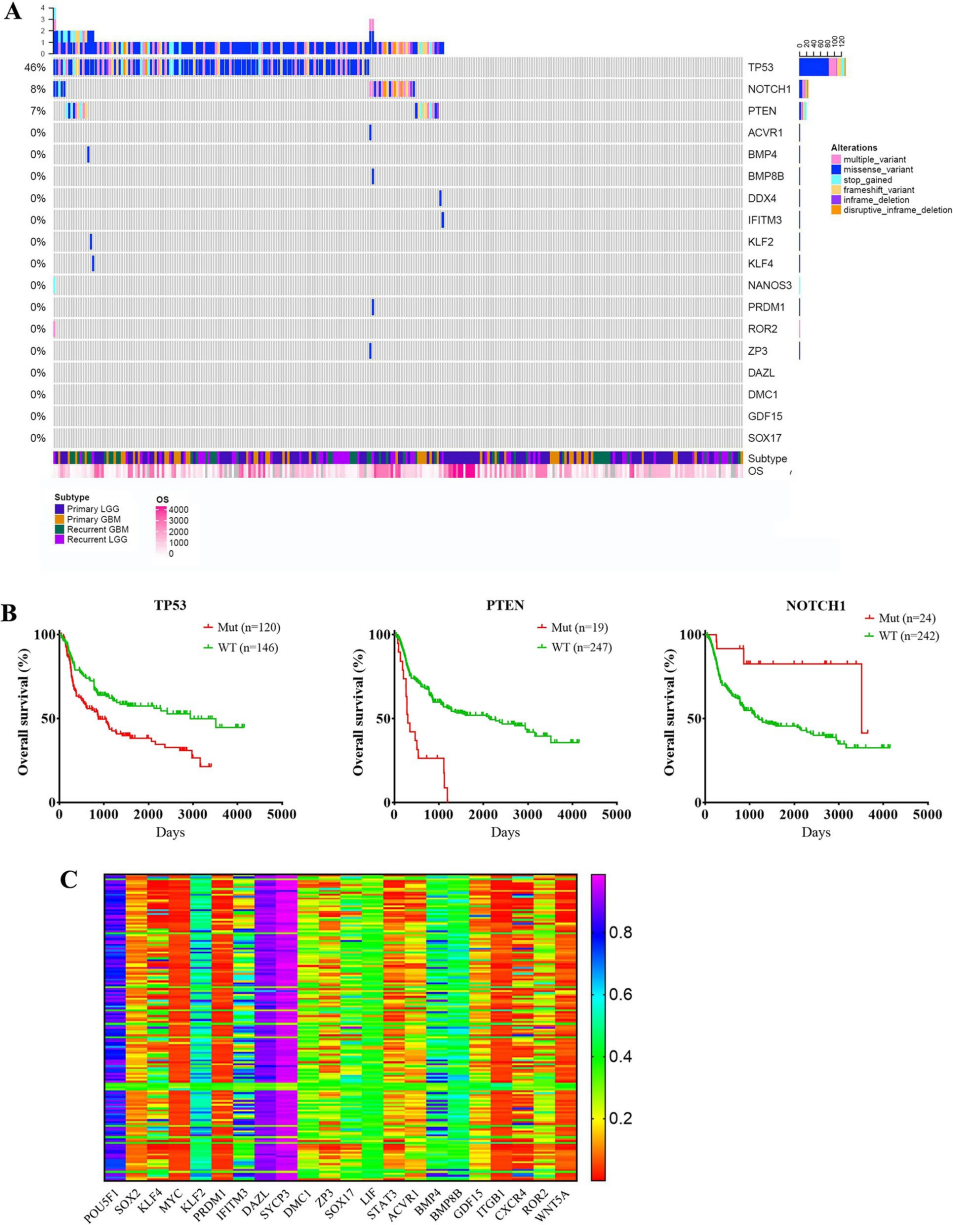
Genetic and epigenetic changes in genes related to embryonic/germ cell development. **A** Whole-exome sequencing data showing the mutational landscape of various genes (the results of *POU5F1, SOX2, STAT3, LIF, ITGB1, CXCR4, WNT5A, SYCP3* and *DMC1* were not found in the CGGA database). **B** Overall survival (OS) curve showing the association of DNA changes in *TP53, PTEN* and *NOTCH1* with the outcomes of gliomas. **C** Methylation data showing the methylation state of various genes

To validate our hypothesis, Chinese Glioblastoma Genome Atlas (CGGA) data were analysed. Since tumourigenicity is thought to be the outcome of a series of genetic and epigenetic changes, we first investigated whether genetic and epigenetic changes in the gene groups were present in human gliomas. The whole-exome sequencing results of gliomas from the CGGA database showed that there were few or no genetic changes among a series of core genes and signalling pathways related to the embryonic/germ cell developmental axis in human gliomas (Fig. 2A). Additionally, the methylation analysis results revealed that most of the genes showed reduced levels of methylation (Fig. 2C). Of note, *TP53* (46%), which inhibits reprogramming, PGC-EGC conversion and oocyte development [20, 36, 45, 46]; *PTEN* (7%), which strongly inhibits PGC-EGC conversion [9, 36]; and *NOTCH1* (8%), which promotes pluripotency [36], were frequently detected among the genetic changes (Fig. 2A). *TP53* and *PTEN* mutations were correlated with poor outcomes, while *NOTCH1* mutations were associated with improved outcomes (Fig. 2B). The data indicated that epigenetic changes but not genetic changes, occurred in most of the core genes related to the embryonic/germ cell developmental axis in human gliomas, while the suppressor genes associated with reprogramming or PGC-EGC conversion frequently lost in gliomas with poor outcomes. Moreover, the reduced levels of methylation in the gene groups supported the activation of genes in gliomas. It is possible that the obtaining of embryonic/germ cell-like traits is activated by methylation changes of embryonic/germ cell-related genes.

### Links of the embryonic/germ cell development-like axis to a malignant prognosis

**Fig. 3:**
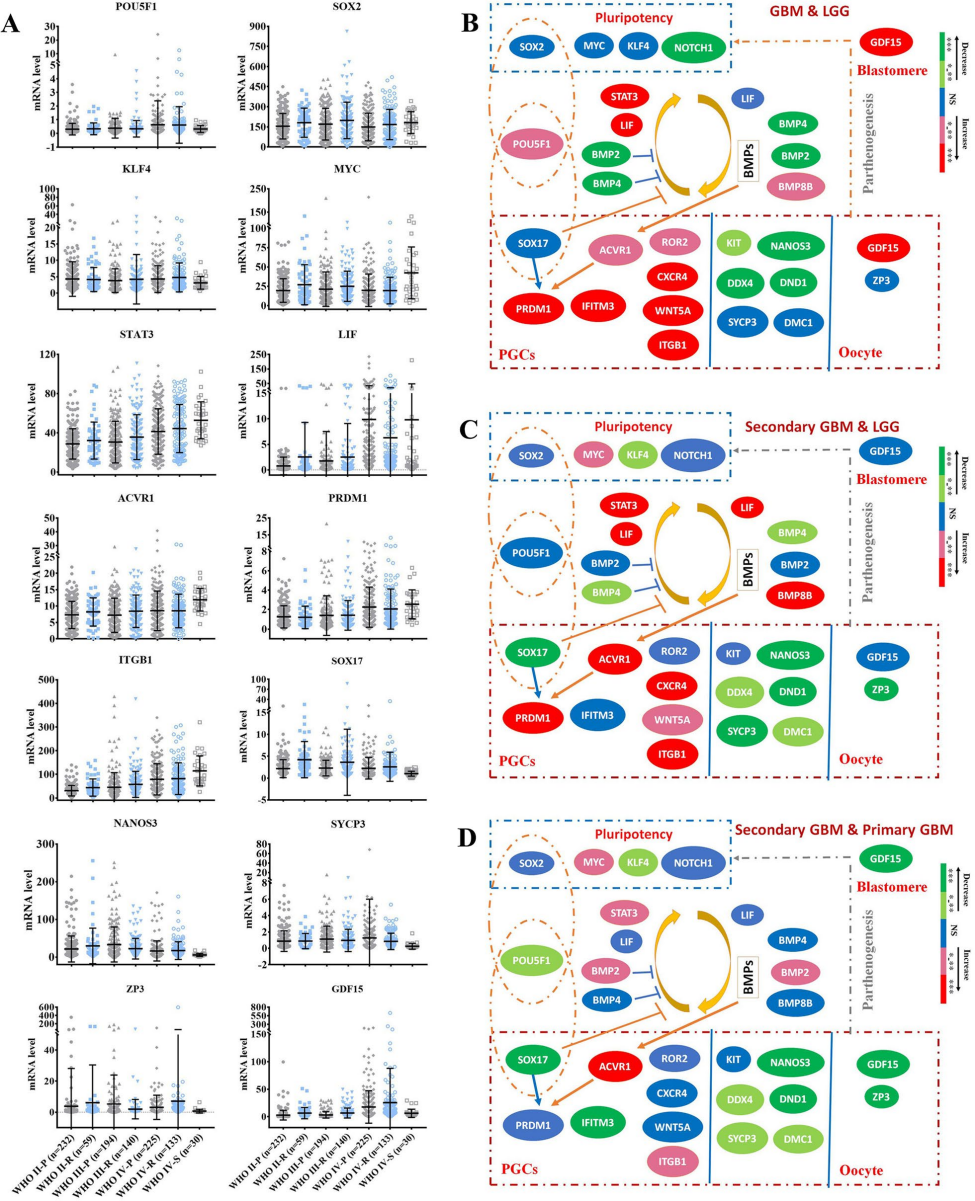
Association between the expression of genes related to embryonic/germ cell development and the pathologic grades/subtypes. **A** RNA sequencing data showing the mRNA expression profile of various genes related to embryonic/germ cell development in subtypes of gliomas that had different pathologic grades. Differentiation of the mRNA levels of embryonic/germ cell-related genes in glioblastomas (GBM) versus lower-grade gliomas (LGG) (**B**), secondary GBM versus LGG (**C**). secondary GBM versus primary GBM (**D**). *P < 0.05, **P < 0.01, ***P < 0.001, ****P < 0.0001

**Fig. 4:**
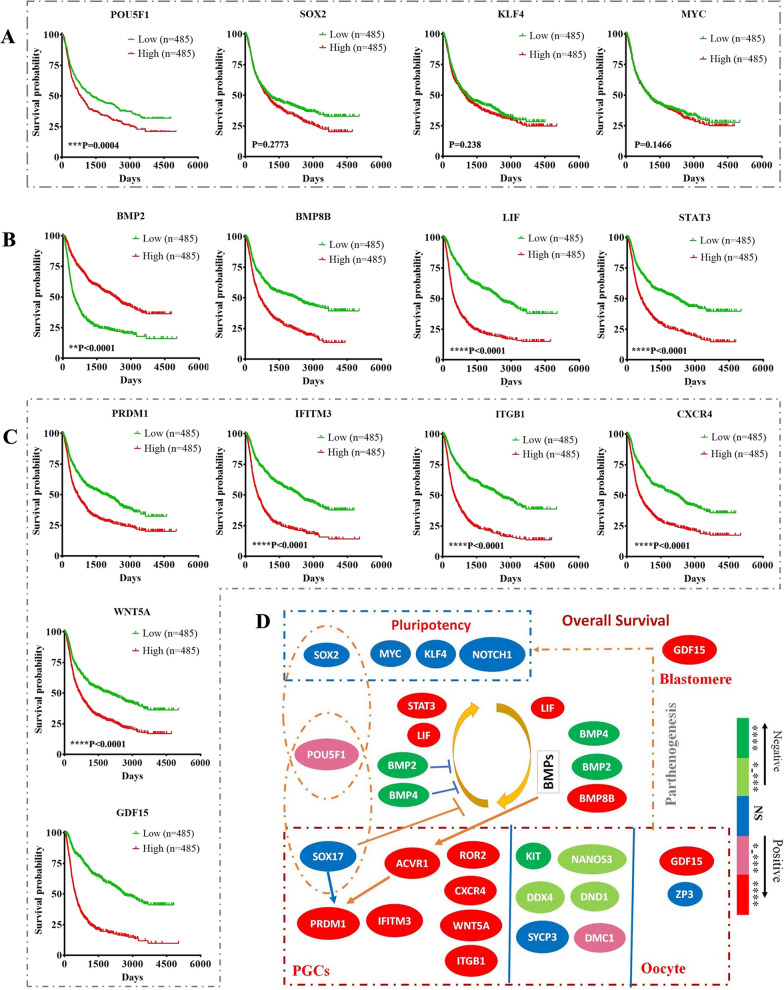
Association between the embryonic/germ cell related gene expression and outcomes of gliomas. RNA sequencing and clinical data showing the mRNA expression profiles of various genes related to IPS reprogramming (**A**) and germ cell development and early blastomere formation (**B**) in gliomas that had different outcomes. **C** Relationship between the mRNA level of embryonic/germ cell-related genes and overall survival of patients. **D** The role of the embryonic/germ cell related gene expression in outcomes of gliomas. *P < 0.05, **P < 0.01, ***P < 0.001, ****P < 0.0001

Gliomas are pathologically classified as glioblastomas (GBM, grade WHO IV) and lower-grade gliomas (LGG, grades WHO II and WHO III) and have distinct malignant stages and outcomes [47]. We analysed the TCGA and GTEx dataset to compare the expression of several genes related with embryonic/germ cell development between gliomas and normal brain tissues. Compared to normal brain tissues, the mRNA level of *SOX2, MYC, NOTCH1, STAT3, BMP2, ACVR1, ITGB1, WNT5A, CXCR4* and *ZP3* increased significantly in both GBM and LGG as well as the mRNA levels of *LIF*, *PRDM1, IFITM3* and *GDF15* increased significantly in GBM but not in LGG (Additional file 1: Fig. S3). However, there was no significant differentiation in the mRNA levels of *BMP4*, *BMP8B, SOX17, KIT, NANOS3, DND1, DAZL, DDX4, ROR2, SYCP3* and *DMC1* between gliomas and normal brain tissues (not shown). To further determine whether the gene groups were activated in gliomas and linked to pathologic classification, malignant prognosis and clinical outcomes, we analysed the mRNA sequencing results of gliomas from the CGGA database. The data showed that IPS reprogramming factors and pluripotency-related genes (*POU5F1, SOX2, KLF4, MYC* and *NOTCH1*) were detected in almost all histologic types and grades of gliomas (Fig. 3A, B, and Additional file 1: Fig. S4A, S5A, Table S3–S5). While the mRNA levels of genes related to pluripotency (*SOX2, KLF4, MYC* and *NOTCH1*) were not obviously correlated with the clinical glioma grade or outcome, the mRNA level of POU5F1, which also plays a crucial role in PGC specification, was often linked to a higher glioma grade and poor outcome (Fig. 3A, B, 4A, C, D and Additional file 1: Fig. S6A, Table S3–S5). These findings revealed that the activation of reprogramming-related genes was commonly seen even in low-grade gliomas; however, it might be insufficient to lead to a malignant prognosis.

As expected, the mRNA sequencing results of gliomas from the CGGA database showed that the mRNA levels of various genes related to germ cell development were also enriched in some gliomas, including *LIF, PRDM1, BMP2, BMP4, BMP8, ACVR1, IFITM3, ITGB1, CXCR4, WNT5A, ROR2, ZP3, GDF15, SOX17, DAZL, DDX4, SYCP3* and *DMC1* (Fig. 3A, and Additional file 1: Fig. S4, S5B, S5C, Table S3, S4). The mRNA levels of *PRDM1*, a core germ cell specification gene, as well as its core upstream gene *ACVR1* and downstream gene *IFITM3* were totally associated with advanced pathologic grades and poor outcomes (Figs. 3B and 4B–D, and Additional file 1: Fig. S5B, S6A, Table S3–S5). High mRNA levels of genes related to PGC proliferation/survival/migration (*ITGB1, CXCR4, WNT5A* and *ROR2*), ES-PGC conversion (*BMP8B* and *LIF*), PGC-EGC conversion (*LIF* and *STAT3*), PGC survival (*DAZL*) and oocytes/early embryos (*GDF15*) also showed totally correlations with an advanced pathologic grade and poor overall survival of patients (Figs. 3B and 4B–D, and Additional file 1: Fig. S5B, S6A, Table S3–S5). Notably, *POU5F1* partners with *SOX2* in human ES cells and partners with *SOX17* in human PGCs [33, 38]. Although the mRNA levels of *SOX17*, a core germ cell specification gene [38], were enriched in some gliomas, they did not show obvious correlations with advanced pathologic grades and poor outcomes (Figs. 3B and D and 4C, D and Additional file 1: Fig. S5B, S6A, Table S3–S5), likely because SOX17 also inhibits PGC-EGC reprogramming [33]. The mRNA level of gene related with later meiosis (*DMC1*) was slightly associated with poor overall survival (Fig. 4C, D and Additional file 1: Fig. S5B, S6A, Table S5). The mRNA levels of genes related to the activation of ES-PGC conversion but inhibition of PGC-EGC conversion (*BMP2* and *BMP4*), late PGCs (*DDX4*), meiosis entry (*SYCP3*) as well as genes related to PGC fate determination (*KIT, NANOS3* and *DND1*) showed no or negative correlations with advanced pathologic grades and poor outcomes (Figs. 3B and 4C and Additional file 1: Fig. S5B, S6A, Table S3–S5). These data suggest that tumour cells arrested in the germ cell-like developmental stage (not early PGC-like stage) and that lost the ability to return to the embryonic cell-like state might not lead to a malignant prognosis of gliomas. Compared with LGG, GBM showed higher expression of *POU5F1, PRDM1, BMP8B, LIF, STAT3, ACVR1, IFITM3, ITGB1, CXCR4, WNT5A, ROR2* and *GDF15* (Fig. 3A, B, and Additional file 1: Fig S2A, Table S4), indicating that both the activation of the PGC-like state and the return of germ cells to the embryo-like state were essential in the malignant behaviours of some gliomas via somatic parthenogenetic embryo-like cycle and/or somatic PGC-EGC/ES-like cycle. Compared with LGG, secondary GBM arising from LGG [48] showed higher expression of *PRDM1, BMP8B, ACVR1, LIF, STAT3, ITGB1, WNT5A, CXCR4, MYC* but decreased expression of genes related to germ cell fate, including *Nanos3, DND1, BMP2, BMP4, SOX17, DDX4, SYCP3, DMC1* and *ZP3* (Fig. 3A, C, and Additional file 1: Fig. S4A, Table S4), indicating that the PGC-EGC/ES-like conversion pathway rather than the mature development pathway might be activated in secondary GBM and that the activation of PGC-EGC/ES-like cycle was linked to the malignant prognosis of LGG. Compared with primary GBM, secondary GBM showed higher expression of *ITGB1, ACVR1, MYC, STAT3* and *BMP2* but significantly decreased expression of genes related to germ cell mature (*Nanos3, DND1, DDX4, SOX17, SYCP3, DMC1, ZP3* and *GDF15*) and genes related to inhibition of PGC-EGC conversion (*BMP4* and *SOX17*) (Fig. 3A, D, and Additional file 1: Fig. S4A, Table S3), further indicating that the activation of PGC-EGC/ES-like conversion was one of driving events to malignant prognosis of gliomas. In addition, the decreased expression of genes related to germ cell development was accompanied by the decreased expression of *GDF15*, consistent with the fact that activation of gene *GDF15* links to the oocyte/early embryo-like state (Fig. 3A, D, and Additional file 1: Table S3). Collectively, these findings indicated that the genes related to the activation of the parthenogenetic embryo-like cycle and activation of the PGC-EGC-like cycle were both linked to poor patient outcomes, which might represent two pathways that drive the malignant prognosis of gliomas.

### Inhibition of embryonic/germ cell cycle-related gene expression among gliomas with 1p19q codeletion

**Fig. 5:**
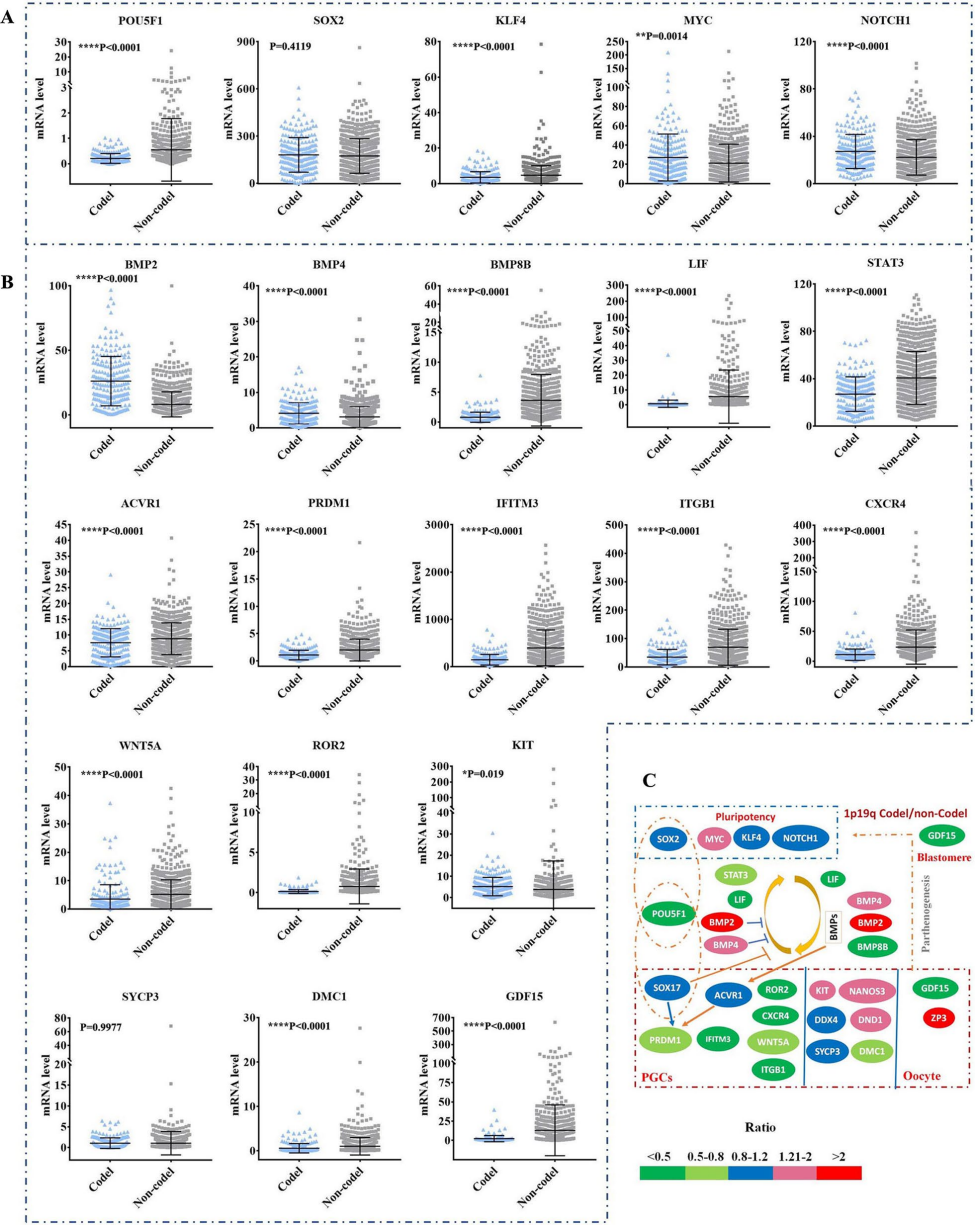
Association between the mRNA levels of various genes and 1p/19q codeletion status in gliomas. RNA sequencing and clinical data showing that the relationship between mRNA levels of genes related to IPS reprogramming and 1p/19q codeletion status (**A**); between mRNA levels of genes related to germ cell development and early blastomere formation and 1p/19q codeletion status (**B**). **C** The ratio of gliomas with 1p/19q codeletion to gliomas without 1p/19q

LGG have wide survival ranges, from 1 to 15 years, and distinct therapeutic sensitivities [47]. LGG with deletion of chromosome arms 1p and 19q (1p19q codeletion) are often associated with impressive therapeutic sensitivities and favourable clinical outcomes [47]. Consequently, we determined whether genes related to the embryonic/germ cell developmental axis were inhibited in glioma samples with 1p19q codeletion. The combined mRNA sequencing data from the 325 and 694 datasets of CGGA showed that the mRNA levels of *POU5F1, LIF, BMP8B, ROR2, CXCR4, IFITM3, ITGB1* and *GDF15* were extremely low in almost all glioma samples with 1p19q codeletion (1p19q-codel) compared to glioma samples without 1p19q codeletion (1p19q-noncodel) (Fig. 5A–C, and Additional file 1: Table S6). Overall, the mRNA levels of *ACVR1, STAT3, WNT5A, KLF4* and *DMC1* were obviously decreased in glioma samples with 1p19q codeletion compared to those in glioma samples without 1p19q codeletion (Fig. 5A–C, and Additional file 1: Fig. S7, Table S6). However, 1p19q codeletion did not inhibit the mRNA levels of *SOX2, SOX17, SYCP3, MYC, ZP3, NOTCH1, DDX4, KIT, NANOS3, DND1, BMP2* and *BMP4* (Fig. 5A–C, and Additional file 1: Fig. S7, Table S6). These findings indicated that 1p19q codeletion robustly inhibited genes involved in promoting the embryonic/germ cell cycle.

### Clinical significance of embryonic/germ cell cycle-related molecular groups among gliomas

**Fig. 6:**
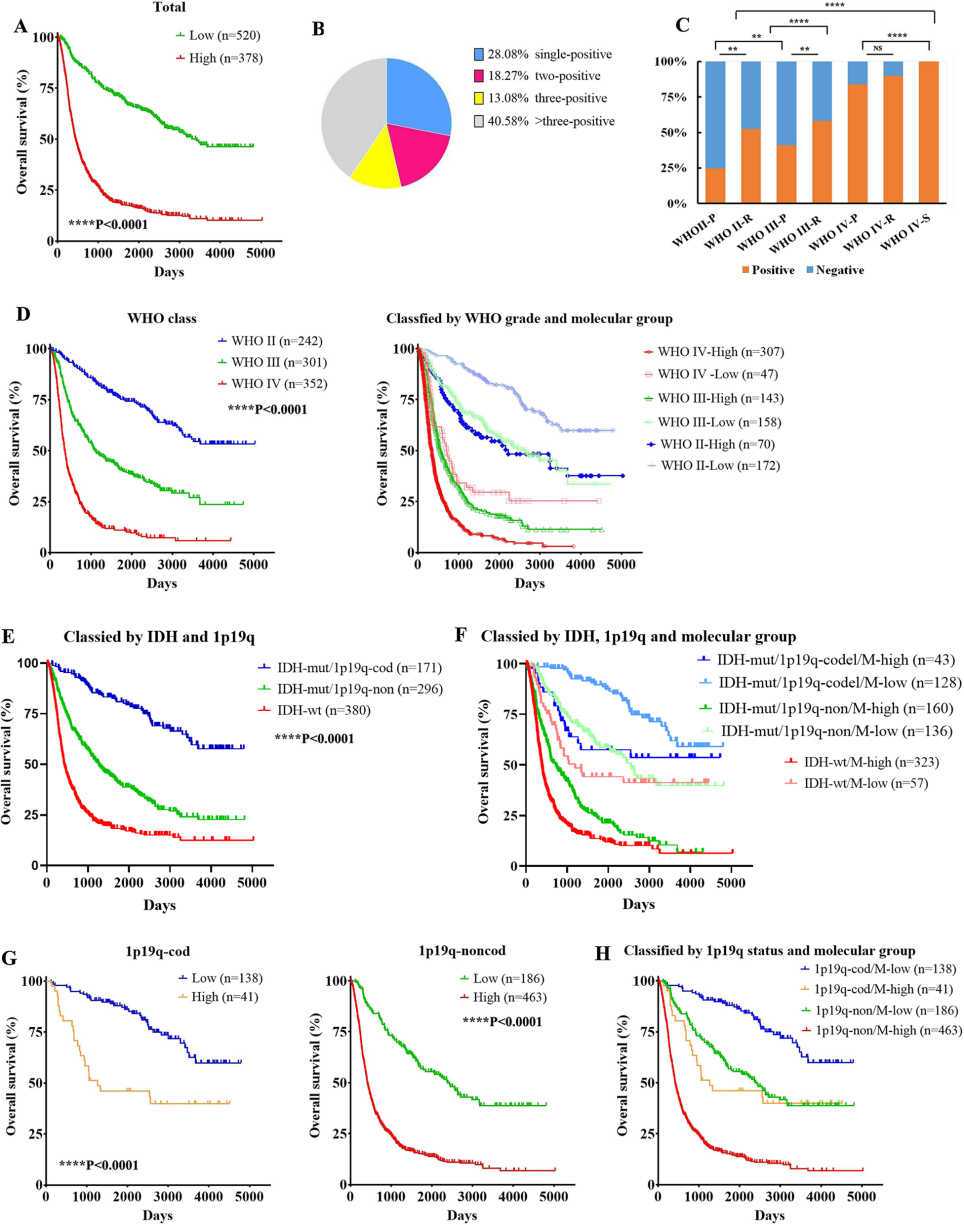
Clinical significance and prevalence of molecular groups among gliomas. **A** Patients separated by the mRNA level of genes in the gene group showed different overall survival. **B** Distribution of gliomas with high expression of single to multiple genes in molecular groups among gliomas with higher expression of genes in molecular groups. **C** Prevalence of the molecular groups among distinct pathological grades. **D** Gliomas classified according to the WHO grade or the WHO combined with the molecular group showed different overall survival. **E** Gliomas classified according to the status of IDH and 1p19q. **F** Patients with the same IDH and 1p19q status classified by the molecular group showed different overall survival. **G** Patients with the same 1p19q status classified by the molecular group showed different overall survival. **H** Gliomas classified according to 1p19q status and the molecular group showed different overall survival

Histopathological classification is often performed in gliomas; however, this method is not sufficient to predict clinical outcomes [47]. Consequently, we performed an analysis of the molecular group related to the embryonic/germ cell cycle (including *LIF, STAT3, PRDM1, IFITM3, ACVR1, CXCR4, WNT5A, ROR2, ITGB1, POU5F1, GDF15* and *BMP8B*) (Additional file 1: Table S7) to determine whether we could identify glioma outcomes more accurately based on the germ cell-related molecular group than based on the histologic class. The combined mRNA sequencing data of 325 and 694 CGGA datasets showed that patients with higher expression of genes in the molecular group had much poorer overall survival than patients with lower expression of genes in the gene groups (Fig. 6A). The median survival of patients in the two groups was 443 days (high) and 3,411 days (low) respectively (Fig. 6A, and Additional file 1: Table S8). Notably, increased expression of genes in the molecular group was frequently observed in the same gliomas (Fig. 6B). Approximately 71.92% of gliomas with increased expression of genes in the molecular group had at least two genes with increased expression (Fig. 6B). Among grade II-primary, II-recurrence, III-primary, II-recurrence, IV-primary, IV-recurrence gliomas and IV-secondary gliomas, the total ratio of gene groups with high expression of a single gene was ~ 24.71%, ~ 52.50%, ~ 40.88%, ~ 58.3%, ~ 83.98%, ~ 89.91% and ~ 100%, respectively (Fig. 6C, and Additional file 1: Table S9), further indicating that activation of the embryonic/germ cell-like developmental axis may be associated with the pathological grade, recurrence and progression of gliomas. Interestingly, the mRNA profiles of the molecular group could be used to separate poor outcomes from improved outcomes among patients who harboured gliomas with the same WHO grade, especially among the patients with grade III gliomas (Fig. 6C, and Additional file 1: Table S8, S10). Median survival was 344 days (higher) and 710 days (lower) among patients with grade IV gliomas, 544 days (higher) and 2633 days (lower) among patients with grade III gliomas, and 2,219 days (higher) and more than 5,000 days (lower) among patients with grade II gliomas (Fig. 6D, Additional file 1: Fig. S8 and Table S8). Notably, glioma classification based on the WHO grade and molecular group could predict clinical behaviours more accurately (Fig. 6E, Additional file 1: Fig. S9A and Table S8, S10). Subtype grade II gliomas with lower expression of genes in the molecular group had favourable clinical outcomes (Fig. 6D, Additional file 1: Fig. S8, and Table S8). Grade III gliomas with higher expression of genes in the molecular group were similar to grade IV gliomas in terms of clinical outcomes (Fig. 6D, Additional file 1: Fig. S8, and Table S8).

In the revised WHO classification of 2016, combining the IDH and 1p19q status serves as a diagnostic molecular biomarker for gliomas. Then we combining the IDH and 1p19q status subdivided gliomas into three groups, including IDH mutant with 1p19q codeletion, IDH mutant without 1p19q codeletion and IDH wildtype. Among the gliomas with IDH mutant and 1p19q codeletion, IDH mutant and 1p19q non-codeletion as well as IDH wildtype in the combined dataset, the median survival of the three groups of patients was > 5000 days, 1258 days and 420 days (Fig. 6E and Additional file 1: Table S11) respectively. Combining the IDH and 1p19q status together with our molecular group could subdivided gliomas into six groups. Among the gliomas with IDH mutant and 1p19q codeletion in the combined dataset, the median survival of the two groups of patients was both > 5000 days (Fig. 6F and Additional file 1: Table S8). Among the gliomas with IDH mutant and 1p19q non-codeletion in the combined dataset, the median survival of the two groups of patients was 723 days (higher) and 2552 days (lower) (Fig. 6F and Additional file 1: Table S8) respectively. Among the gliomas with IDH wildtype in the combined dataset, the median survival of the two groups of patients was 387 days (higher) and 1196 days (lower) (Fig. 6F and Additional file 1: Table S8) respectively.

We then used 1p19q status together with our molecular group to classified the gliomas. Among patients who had gliomas with 1p19q codeletion in the combined dataset, patients with higher expression of genes in the gene group (median survival = 1265 days) had much poorer overall survival than patients with lower expression of genes in the molecular group (median survival > 5000 days) (Fig. 6G and Additional file 1: Table S8). Among the gliomas without 1p19q codeletion in the combined dataset, the median survival of the two groups of patients was 415 days (higher) and 2382 days (lower) (Fig. 6H and Additional file 1: Table S8) respectively. Classification by combining the 1p19q status and the molecular group could subdivide gliomas into four subtypes with distinct clinical outcomes (Fig. 6H, and Additional file 1: Table S8), suggesting a possible molecular method to predict clinical behaviour. These findings indicate that the embryonic/germ cell cycle-related molecular group can be used as a good marker for predicting clinical outcomes and may be associated to the low-glioma progression to advanced gliomas.

## Appearance of embryonic/germ cell-like cells in gliomas

**Fig. 7:**
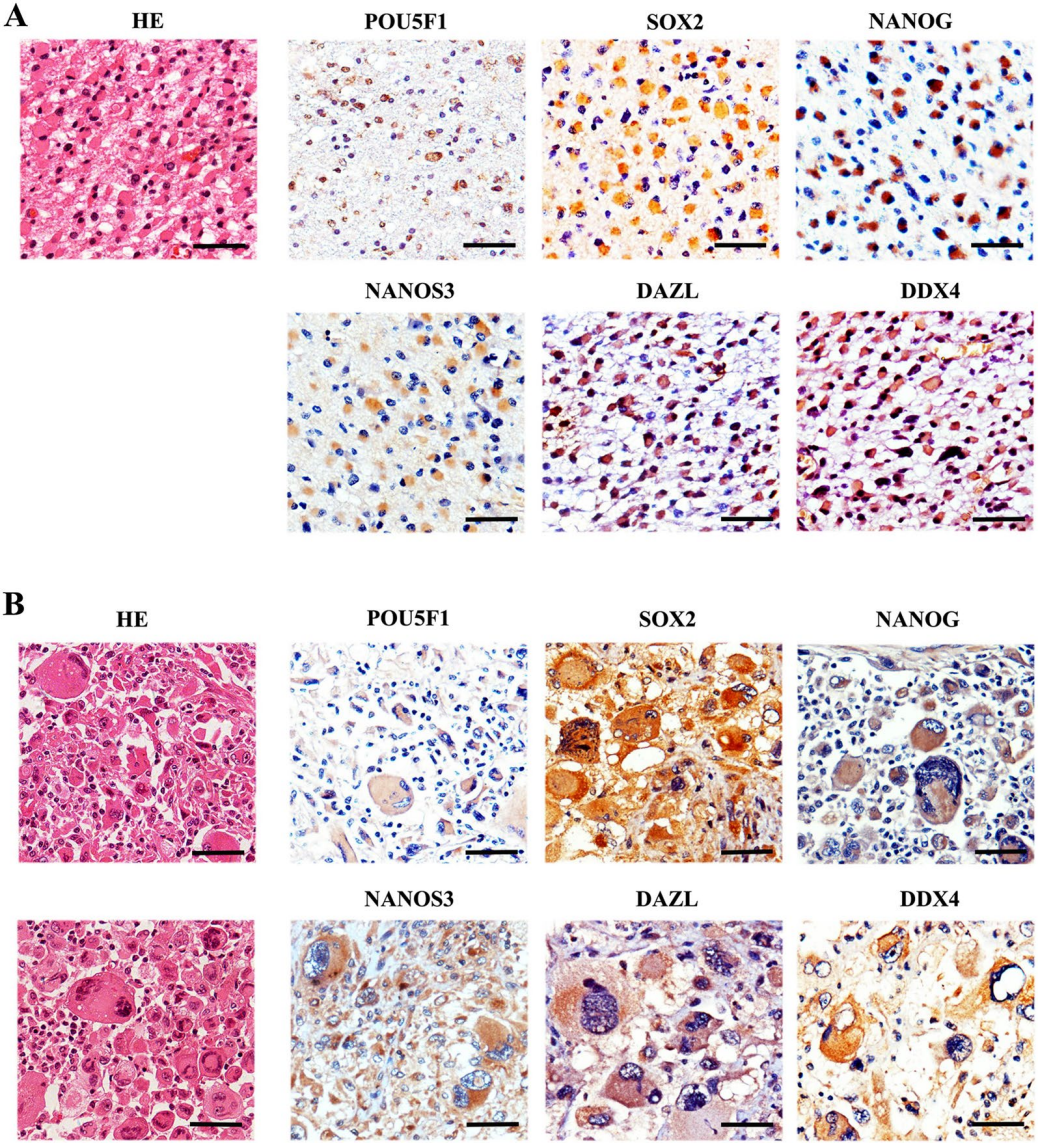
Appearance of germ cell-like cells in human glioma tissues. **A** Immunohistochemistry assays showing the expression and colocalization of the indicated proteins in lower-grade gliomas. **B** Immunohistochemistry assays showing the expression and colocalization of the indicated proteins in grade IV gliomas. Scale bar = 50 μm

**Fig. 8:**
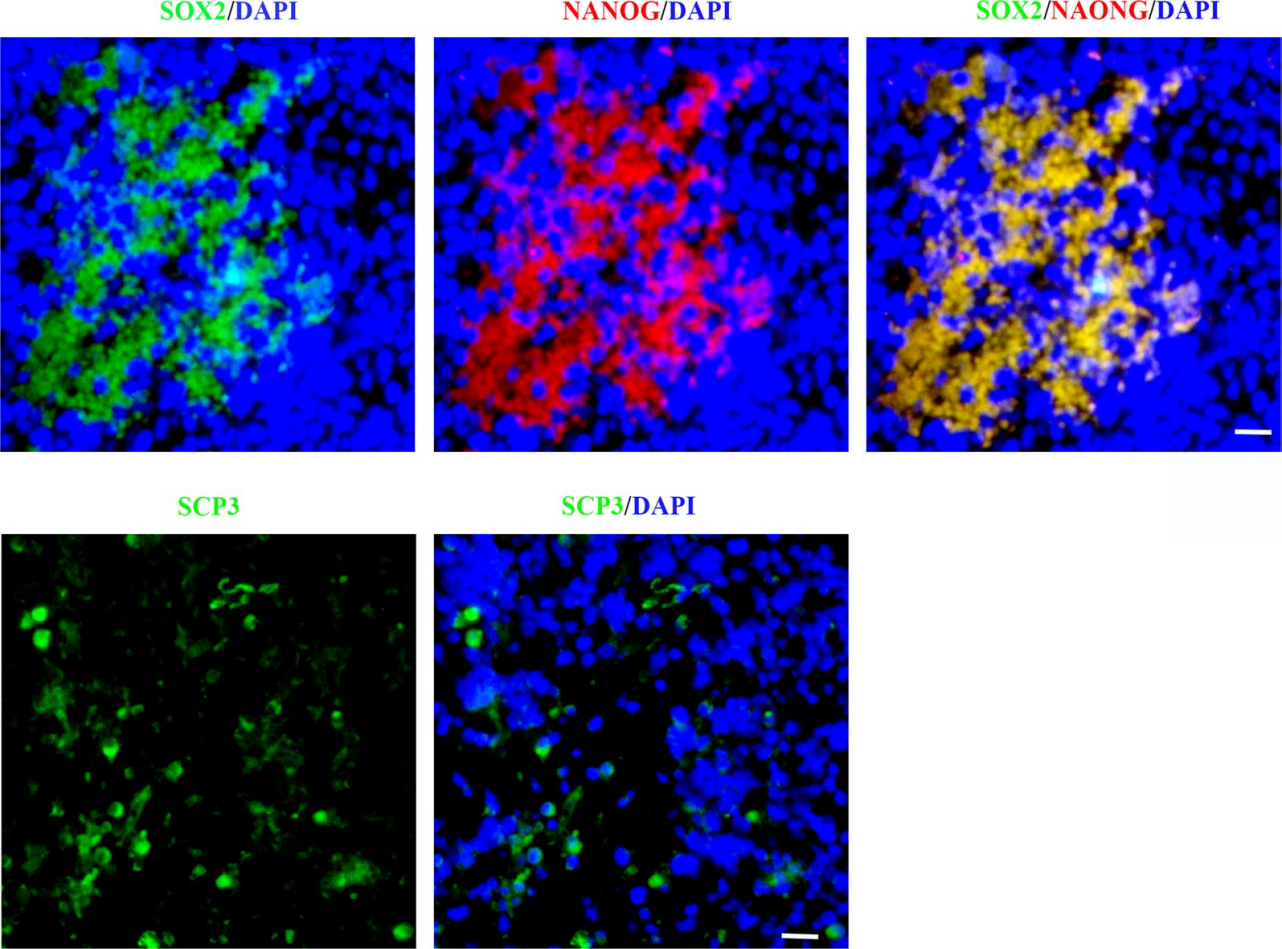
Immunofluorescence assays of embryonic/germ cell-related protein in human glioma tissues. **A** Co-staining of embryonic/germ cell-related protein in human glioma tissues. **B** Immunofluorescence assays showed the expression of meiosis marker in human glioma tissues. Scale bar = 25 μm

We then investigated whether embryonic/germ cell-like cells were present in human gliomas and linked to malignant traits. HE and immune staining showed that germ cell-like cells could be observed in some human glioma tissues, and the appearance of germ cell-like cells was associated with the pathological grade of gliomas (Figs. 7 and 8 and Additional file 1: Fig. S9). Compared with LGG, germ cell-like cells were easily observed in GBM (Additional file 1: Table S12). Notably, a series of embryonic/germ cell-like cells at different developmental stages could be observed in the same tumour tissues among some grade IV gliomas (approximately 40%), including PGC-like cells, oocyte-like cells and parthenogenetic preimplantation embryo-like cells, indicating that a somatic parthenogenetic embryo-like cycle might be present in some GBM (Fig. 7B, and Additional file 1: Fig. S9). It was documented that polyploid giant cancer cells (PGCCs) were blastomere-like cells [1, 49]. Taken together, it is possible that activation of the embryonic/germ cell-like developmental axis occurs during tumour initiation and malignant prognosis.

## Discussion

Our findings showed that germ cell-like cells were present in cultured glioma cells and glioma tissues and that deletion of *DAZL* repressed both PGC-like cell formation and tumour initiation in human glioma cell lines. Moreover, the mRNA sequencing results showed that a series of genes related to reprogramming, PGC specification and germ cell development could be detected in most gliomas. Together with reports that embryonic/germ cell-specific genes are essential in malignant tumour behaviours [17–27, 50], it is possible that activation of the embryonic/germ cell-like state may be a necessary precondition for glioma development, which can explain why gliomas show high embryonic/germ cell traits and extensively express genes related to embryonic/germ cell development and testis antigens of tumours [2, 5, 15, 16, 24, 26].

However, our findings indicated that reprogramming activation or germ cell-like state activation alone seemed to be insufficient to lead to a malignant prognosis. In contrast, the increased mRNA levels of genes related to the activation of the embryonic/germ cell-like cycle showed strong correlations with malignant prognoses and poor clinical outcomes, possibly because this cycle could lead to tumours with migratory PGC-like cells (which are linked to metastasis) and a naive ES/PGC-like state (which is linked to tumourigenicity) through the somatic parthenogenetic embryo-like cycle or somatic PGC-EGC-like cycle. Furthermore, committed PGC state which was induced by DAZL from migratory PGCs (uncommitted state) was hardly reversed to ES state [34, 35]. Consistent with this, high expression of the genes associated with PGC fate determination usually did not lead to poor outcomes of gliomas, such as *KIT, DND1, NANOS3* and *DDX4* [34–37]. Notably, it was shown that some core oncogenes/tumour suppressors strongly upregulated/repressed IPS reprogramming (*TP53, RB, MYC*) [45], PGC-like cell formation (*TP53*), PGC-EGC conversion (*TP53, PTEN*) [9], meiosis (*TP53*) [51] and primary oocyte maturation (TP53) [20, 52], which further supported the essential role of the embryonic/germ cell-like cycle in malignant tumour prognosis [53]. The *TP53* or *PTEN* mutation associated with aggressive gliomas were also documented [5, 54–56]. *TP53* or *PTEN* mutation sites could cause different gain/loss of function. Our previous study [20] and some documents showed *TP53* or *PTEN* deletion [9, 45] would promote the embryonic/germ cell-like cell formation. Whether different *TP53* or *PTEN* mutations could cause the similar re-activation of embryonic/germ cell traits is not clear. It will be interesting to explore this possibility in the future.

PGC-like cells and GBM stem-like cell populations might be different but have the intimate link. In fact, many germ cell markers were used to identify cancer stem cells, such as *SSEA1*, *CD117, Sox2, PRDM14* [16, 18, 22, 25]. Especially, *SSEA1*, a marker of PGCs, was a general marker of tumour initiating cells in human glioblastoma [16], which provied the close link between GBM stem-like cell populations and PGC-like cells. In our views, the GBM stem-like cells might represent one of status of embryonic/germ cell axis, resembling early PGC-like population. Distinct form cancer stem cell concept, our data indicated that it is possible that the real cancer stemness is not the “cancer stem cells” but the whole embryonic/germ cell-like developmental axis including a serial of developmental status, such as embryonic stem-like cells, PGC-like cells, late germ cells and early blastomere-like cells [53].

In summary, our findings indicated that a crucial role of germ cell-like cell formation in glioma initiation as well as activation of the parthenogenetic embryo-like cycle and PGC-EGC-like cycle might represent two driving events in the malignant prognosis of gliomas, which may provide a novel way to better understand the nature of glioma and develop targeted therapies for gliomas as well as important markers for predicting clinical outcomes in gliomas.

## Supplementary Information

**Additional file 1: Figure S1. **PCR showed the DAZL withheterozygote (arrow). **Figure S2.** (A) Immunofluorescence assays showed the expression of DAZLin cultured A172^mut^ cells. (B) AP staining showing PGC-like cells inA172^mut^ glioma cells.  (C) Thecomparison of indicated genes between A172 bottom cells and A172 upper cells Scale bar=25 μm.  **Figure S3.** Increase expression of genes related to embryonic/germ cell developmentin gliomas versus normal brain tissues. *P<0.01. **Figure S4. **Associationbetween the expression of genes related to embryonic/germ cell development andthe pathologic grades/outcomes of gliomas. (A) RNA sequencing showing the mRNA expressionprofiles of the indicated genes in subtypes of gliomas that had different pathologicgrades. (B) RNA array showing the mRNA expression profile of DAZL in subtypesof gliomas that had different pathologic grades. **Figure S5.** Association between the expression of genes related to embryonic/germcell development and the pathologic grades/outcomes of gliomas. (A) RNAsequencing and clinical data showing the mRNA expression profiles of the genesrelated to IPS reprogramming in gliomas that had different pathologic gradesand outcomes. (B) RNA sequencing and clinical data showing the mRNA expressionprofiles of the genes related to IPS reprogramming in gliomas that haddifferent pathologic grades and outcomes. (C) RNA array showing the mRNAexpression profile of DAZL in gliomas that had different pathologic grades. **Figure S6.** Association between the expression of genes related to pluripotency andthe pathologic grades/outcomes of gliomas. (A) RNA sequencing and clinicaldata showing the mRNA expression profiles of the indicated genes in gliomasthat had different pathologic grades and outcomes. **Figure S7. **Associationbetween the mRNA levels of various genes and 1p/19q codeletion status ingliomas.(A) RNA sequencing and clinical data showing the relationship between the mRNAlevel of the indicated genes and 1p/19q codeletion status. (B) The ratio ofgliomas with 1p/19q codeletion to gliomas without 1p/19q codeletion and the meanmRNA values of various genes. **Figure S8. **Clinicalsignificance and prevalence of molecular groups among gliomas. (A) Patients with the same pathological gradeseparated by the mRNA level of genes in the gene group showed different overallsurvival. **Figure S9. **Appearance ofgerm cell-like cells in human glioma tissues. (A) Morphology and marker expression of embryonic/germ cell-like cellsin human glioma tissues.(B) HE stainingand Immunohistochemistry assays showing the germ cell-like celland embryolike structures at different developmental stages. **Table** **S1**. Sequencing data of CRIPSR-Cas9 knockout glioma cells. **Table** **S2**. Detailed information fromthe CGGA database regarding mRNA sequencing. **Table** **S3**. Comparison of geneexpression in subtype of gliomas with distinct WHO classification (Unpaired ttest with Welch's correction). **Table S4.** Comparison of gene expression in gliomas withdistinct WHO classifications (Unpaired t test with Welch's correction and MannWhitney test). **Table** **S5**. Comparison of the mediansurvival of gliomas with distinct gene expression. **Table** **S6**. Comparison of geneexpression in gliomas with distinct 1p19q codeletion statue (Unpaired t testwith Welch's correction). **Table S7.** RNA sequencing cut-offvalues of the genes in the molecular group. **Table S8. **Comparisonof the median survival of gliomas with distinct classifications. **Table S9.** Positive ratio ofmolecular groups in distinct glioma types. **Table** **S10**. Comparison of the median survivalof gliomas with the WHO classification. **Table** **S11**.Comparison of the median survival of gliomas with the WHO classification. **Table S12.** Expression ratio ofproteins related to embryonic/germ cells in glioma tissues.

## Data Availability

The authors confirm that the data supporting the findings of this study are available within the article and its additional materials.

## Abbreviations

CGGA: Chinese Glioblastoma Genome Atlas
EC cells: Embryonal carcinoma cells
EC cells: Embryonal carcinoma cells
EGCs: Embryonic germ cells
ES cells: Embryonic stem cells
iPS cells: Induced pluripotent stem cells
PGCs: Primordial germ cells
GTEx: The Genotype-Tissue Expression
TCGA: The Cancer Genome Atlas
PRDM14: PR/SET Domain 14
PRDM1: PR/SET Domain 1
POU5F1: POU Class 5 Homeobox 1
SOX2: SRY-Box Transcription Factor 2
NANOG: Nanog Homeobox
DAZL: Deleted in Azoospermia Like
DDX4: DEAD-Box Helicase 4
MYC: MYC Proto-Oncogene
BHLH: Transcription factor
KLF4: KLF Transcription Factor 4
NOTCH1: Notch Receptor 1
BMP2: Bone Morphogenetic Protein 2
BMP4: Bone Morphogenetic Protein 4
BMP8B: Bone Morphogenetic Protein 8b
LIF: LIF Interleukin 6 Family Cytokine
SOX17: SRY-Box Transcription Factor 17
ACVR1: Activin A Receptor Type 1
IFITM3: Interferon Induced Transmembrane Protein 3
KIT: KIT Proto-Oncogene, Receptor Tyrosine Kinase
NANOS3: Nanos C2HC-Type Zinc Finger 3
DND1: DND MicroRNA-Mediated Repression Inhibitor 1
ITGB1: Integrin Subunit Beta 1
CXCR4: C-X-C Motif Chemokine Receptor 4
WNT5A: Wnt Family Member 5 A
ROR2: Receptor tyrosine kinase like orphan receptor 2
SYCP3: Synaptonemal Complex Protein 3
DMC1: DNA Meiotic Recombinase 1
TP53: Tumor Protein P53
PTEN: Phosphatase And Tensin Homolog
STAT3: Signal transducer and activator of transcription 3
ZP3: Zona Pellucida Glycoprotein 3
GDF15: Growth differentiation factor 15
