# Supplementary material for: Activation of embryonic/germ cell-like axis links poor outcomes of gliomas
Source: Cancer Cell Int. 2022 Nov 26;22:371. doi: 10.1186/s12935-022-02792-8 (PMC9701408; doi:10.1186/s12935-022-02792-8)
Supplement: Supplementary file 1 — Additional file 1: Figure S1. PCR showed the DAZL withheterozygote (arrow). Figure S2. (A) Immunofluorescence assays showed the expression of DAZLin cultured A172mut cells. (B) AP staining showing PGC-like cells inA172mut glioma cells. (C) Thecomparison of indicated genes between A172 bottom cells and A172 upper cells Scale bar=25 μm. Figure S3. Increase expression of genes related to embryonic/germ cell developmentin gliomas versus normal brain tissues. *P<0.01. Figure S4. Associationbetween the expression of genes related to embryonic/germ cell development andthe pathologic grades/outcomes of gliomas. (A) RNA sequencing showing the mRNA expressionprofiles of the indicated genes in subtypes of gliomas that had different pathologicgrades. (B) RNA array showing the mRNA expression profile of DAZL in subtypesof gliomas that had different pathologic grades. Figure S5. Association between the expression of genes related to embryonic/germcell development and the pathologic grades/outcomes of gliomas. (A) RNAsequencing and clinical data showing the mRNA expression profiles of the genesrelated to IPS reprogramming in gliomas that had different pathologic gradesand outcomes. (B) RNA sequencing and clinical data showing the mRNA expressionprofiles of the genes related to IPS reprogramming in gliomas that haddifferent pathologic grades and outcomes. (C) RNA array showing the mRNAexpression profile of DAZL in gliomas that had different pathologic grades. Figure S6. Association between the expression of genes related to pluripotency andthe pathologic grades/outcomes of gliomas. (A) RNA sequencing and clinicaldata showing the mRNA expression profiles of the indicated genes in gliomasthat had different pathologic grades and outcomes. Figure S7. Associationbetween the mRNA levels of various genes and 1p/19q codeletion status ingliomas.(A) RNA sequencing and clinical data showing the relationship between the mRNAlevel of the indicated genes and 1p/19q codeletion status. (B) The rat [file 12935_2022_2792_MOESM1_ESM.pdf]

Activation of embryonic/germ cell-like axis links poor outcomes of gliomas

Zhan Ma†[1, 2], Fengyu Zhang†[1], Ji Xiong†[3], Haishi Zhang†[4], Hui-Kuan Lin[5], Chunfang Liu[1]*

[1]Department of Laboratory Medicine, Huashan Hospital, Shanghai Medical College, Fudan University, Shanghai, 200040, China.

[2]Department of Laboratory Medicine, Shanghai Children's Hospital, Shanghai Jiao Tong University, Shanghai 200040, China.

[3]Department of Pathology, Shanghai Medical College, Fudan University, Shanghai, 200040, China.

[4]Department of Neurosurgery, Huashan Hospital, Fudan University, Shanghai 200040, China.

[5]Department of Cancer Biology, Wake Forest School of Medicine, Winston-Salem, NC 27157, USA.

†These authors contributed equally.

*Correspondence and requests for materials should be addressed C.L. (chunfang_liu@fudan.edu.cn).

Supplementary Appendix

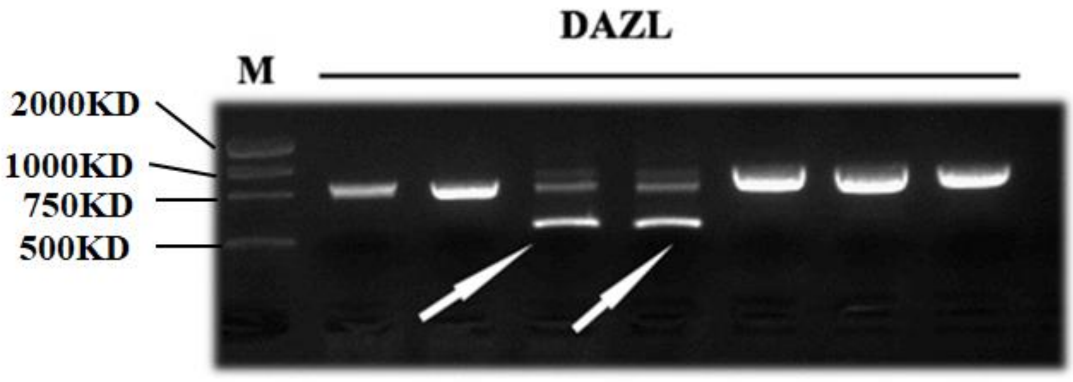

Supplementary Figure S1. PCR showed the DAZL with heterozygote (arrow).

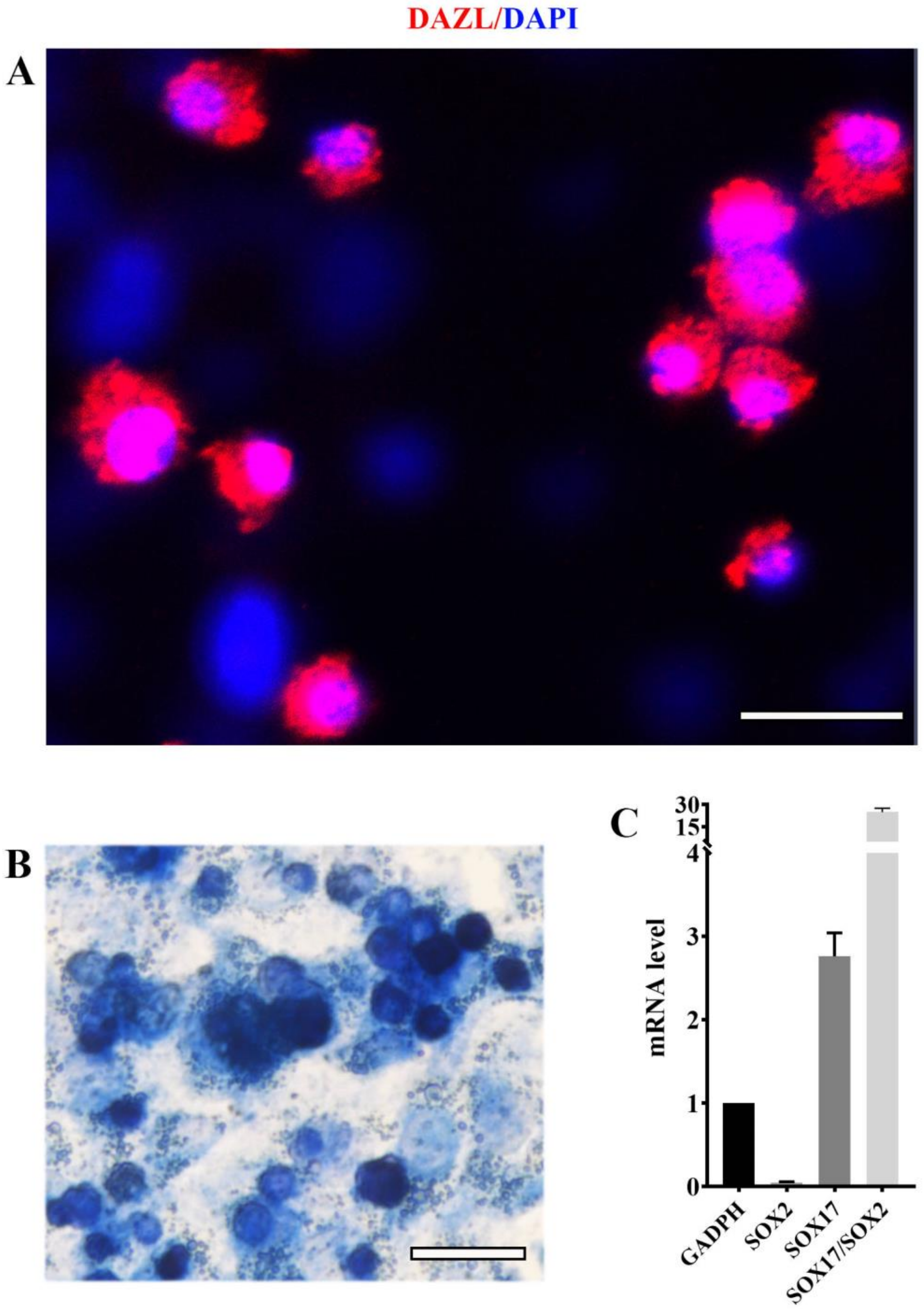

Supplementary Figure S2. (A) Immunofluorescence assays showed the expression of DAZL in cultured A172^{mut} cells. (B) AP staining showing PGC-like cells in A172^{mut} glioma cells. (C) The comparison of indicated genes between A172 bottom cells and A172 upper cells Scale bar=25 μm

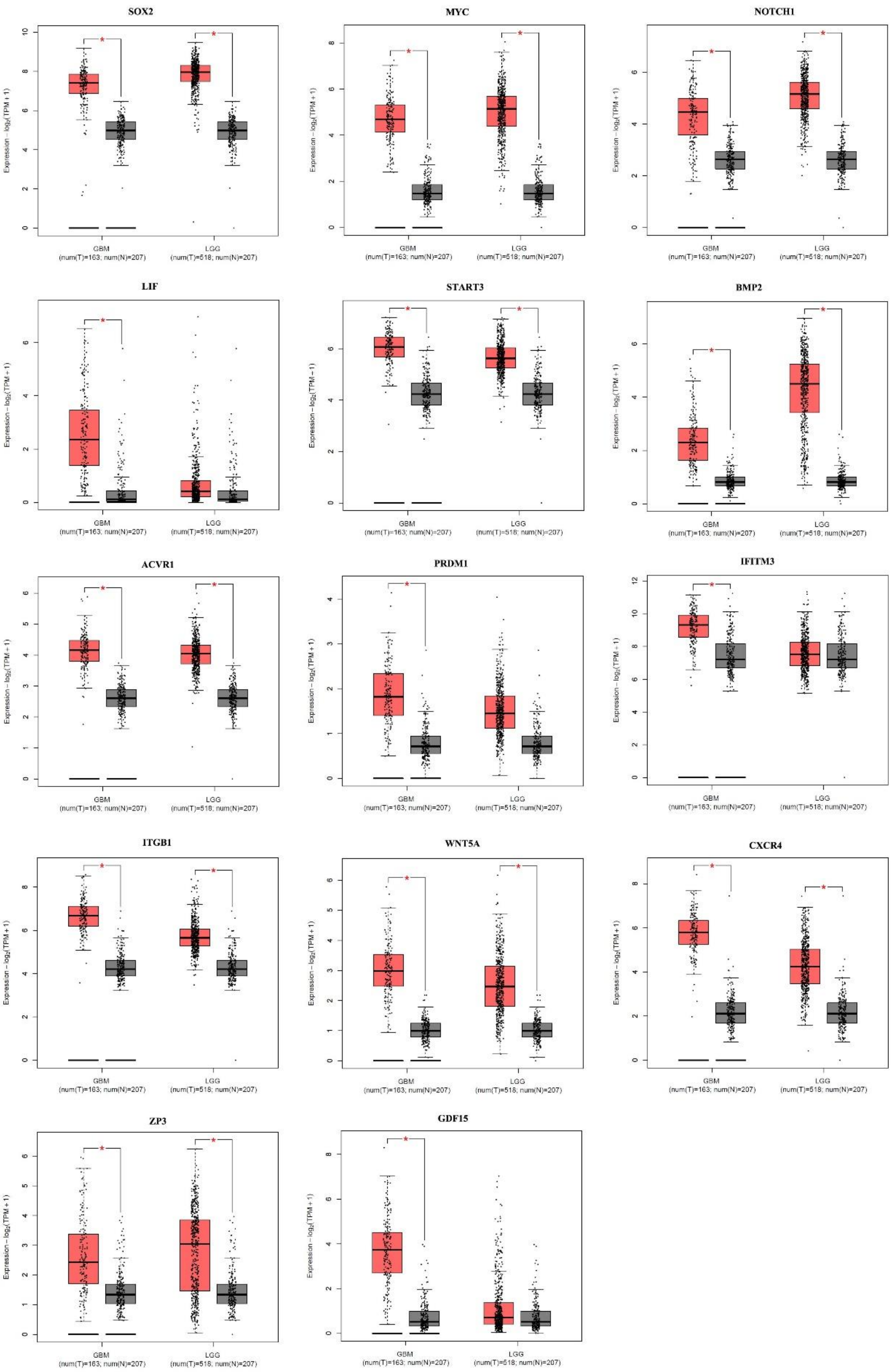

Supplementary Figure S3. Increase expression of genes related to embryonic/germ cell development in gliomas versus normal brain tissues. *P<0.01

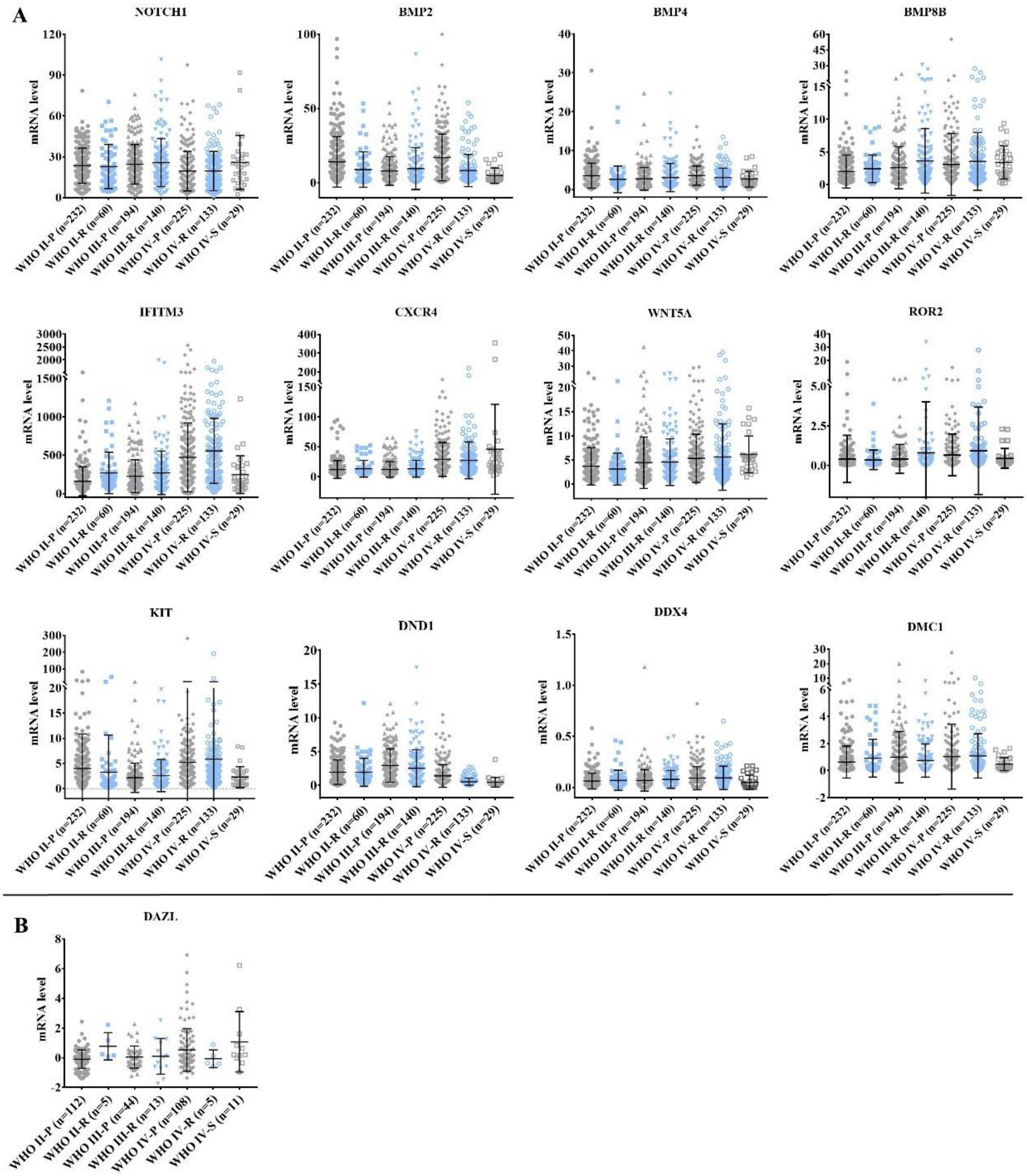

Supplementary Figure S4. Association between the expression of genes related to embryonic/germ cell development and the pathologic grades/outcomes of gliomas. (A) RNA sequencing showing the mRNA expression profiles of the indicated genes in subtypes of gliomas that had different pathologic grades. (B) RNA array showing the mRNA expression profile of DAZL in subtypes of gliomas that had different pathologic grades.

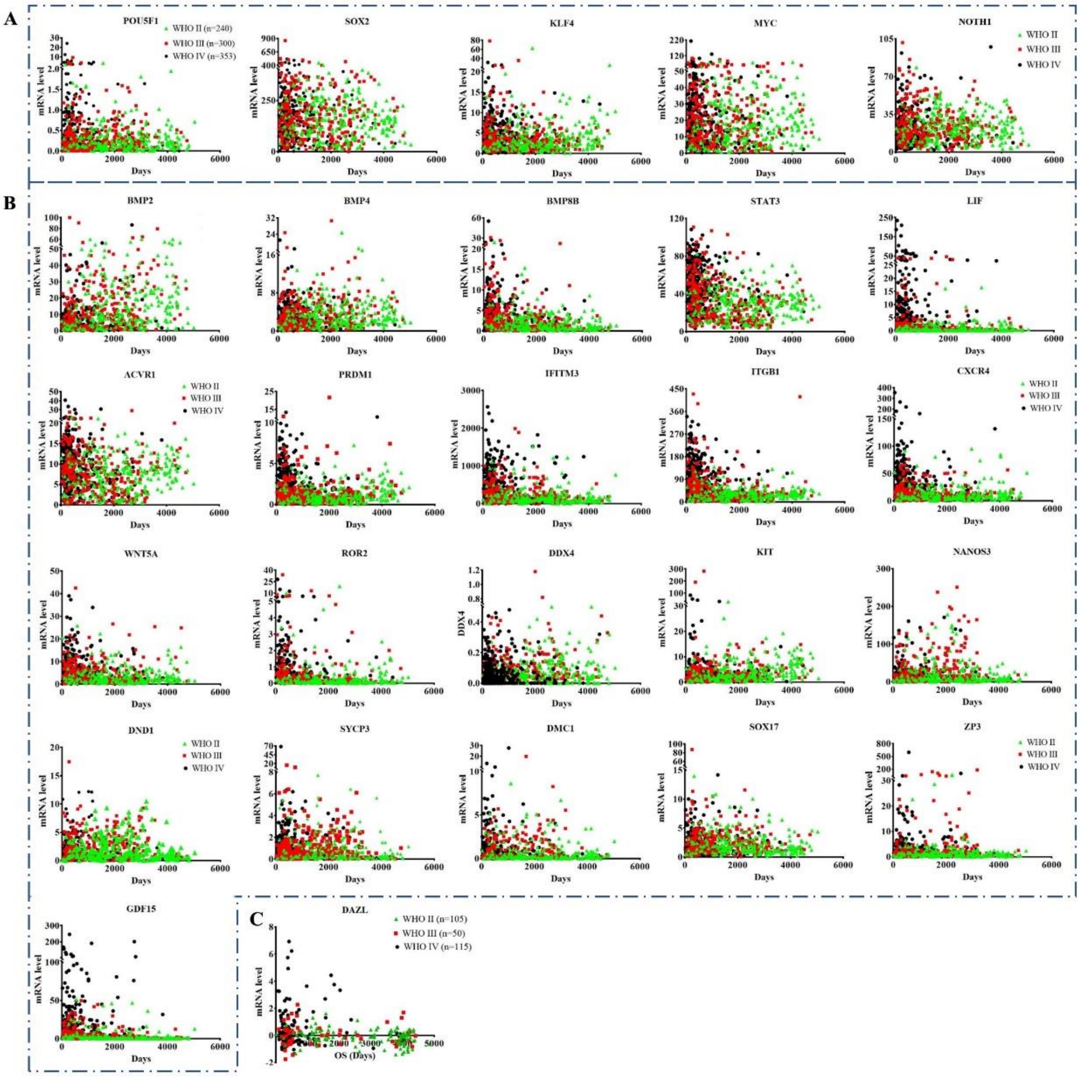

Supplementary Figure S5. Association between the expression of genes related to embryonic/germ cell development and the pathologic grades/outcomes of gliomas. (A) RNA sequencing and clinical data showing the mRNA expression profiles of the genes related to IPS reprogramming in gliomas that had different pathologic grades and outcomes. (B) RNA sequencing and clinical data showing the mRNA expression profiles of the genes related to IPS reprogramming in gliomas that had different pathologic grades and outcomes. (C) RNA array showing the mRNA expression profile of DAZL in gliomas that had different pathologic grades.

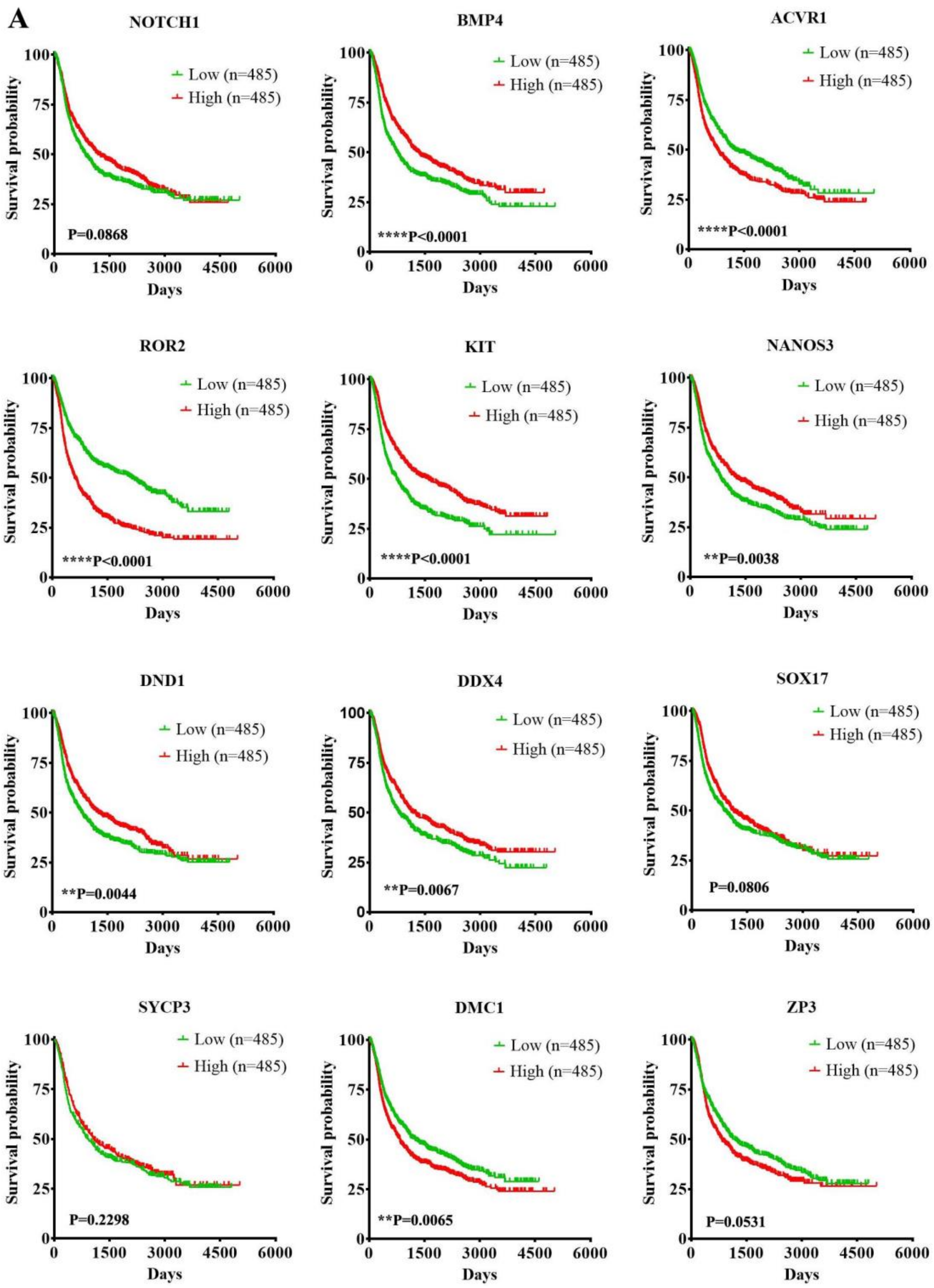

Supplementary Figure S6. Association between the expression of genes related to pluripotency and the pathologic grades/outcomes of gliomas. (A) RNA sequencing and clinical data showing the mRNA expression profiles of the indicated genes in gliomas that had different pathologic grades and outcomes.

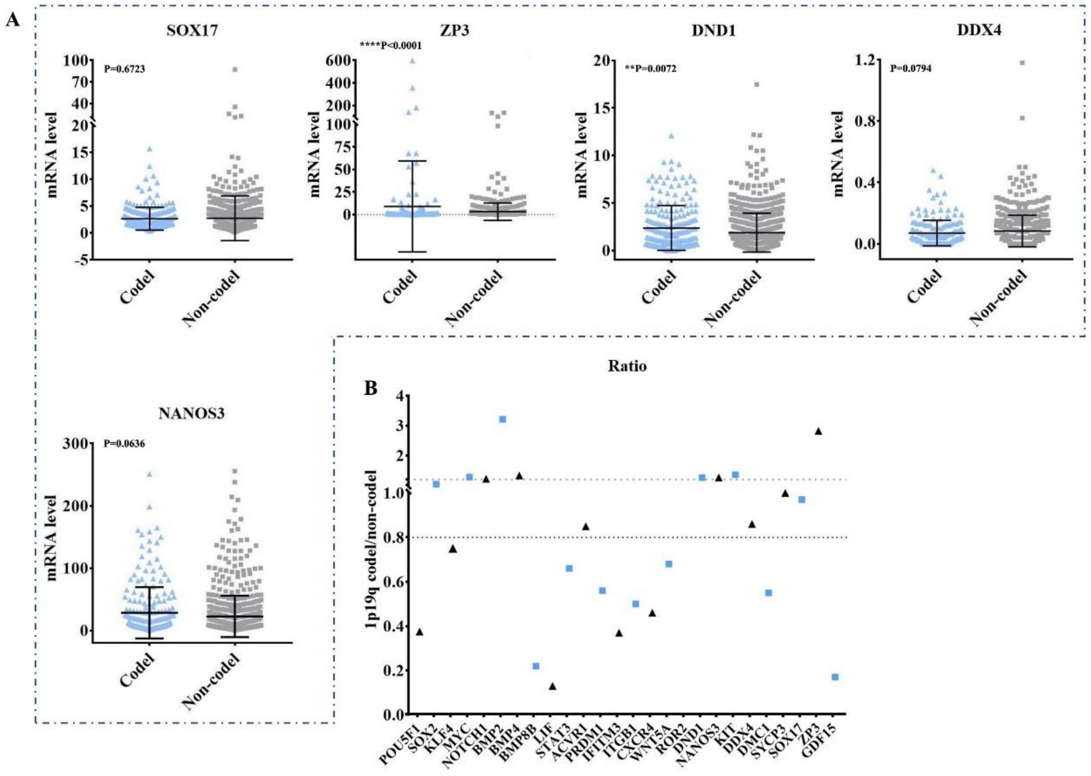

Supplementary Figure S7. Association between the mRNA levels of various genes and 1p/19q codeletion status in gliomas. (A) RNA sequencing and clinical data showing the relationship between the mRNA level of the indicated genes and 1p/19q codeletion status. (B) The ratio of gliomas with 1p/19q codeletion to gliomas without 1p/19q codeletion and the mean mRNA values of various genes.

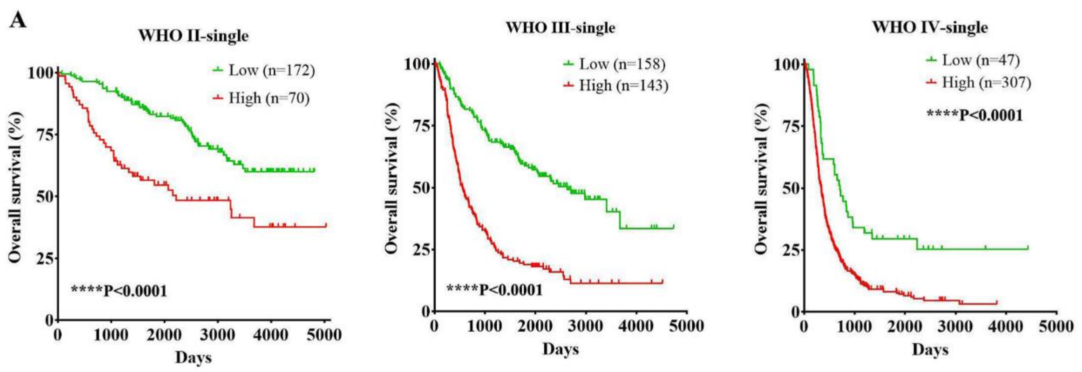

Supplementary Figure S8. Clinical significance and prevalence of molecular groups among gliomas. (A) Patients with the same pathological grade separated by the mRNA level of genes in the gene group showed different overall survival.

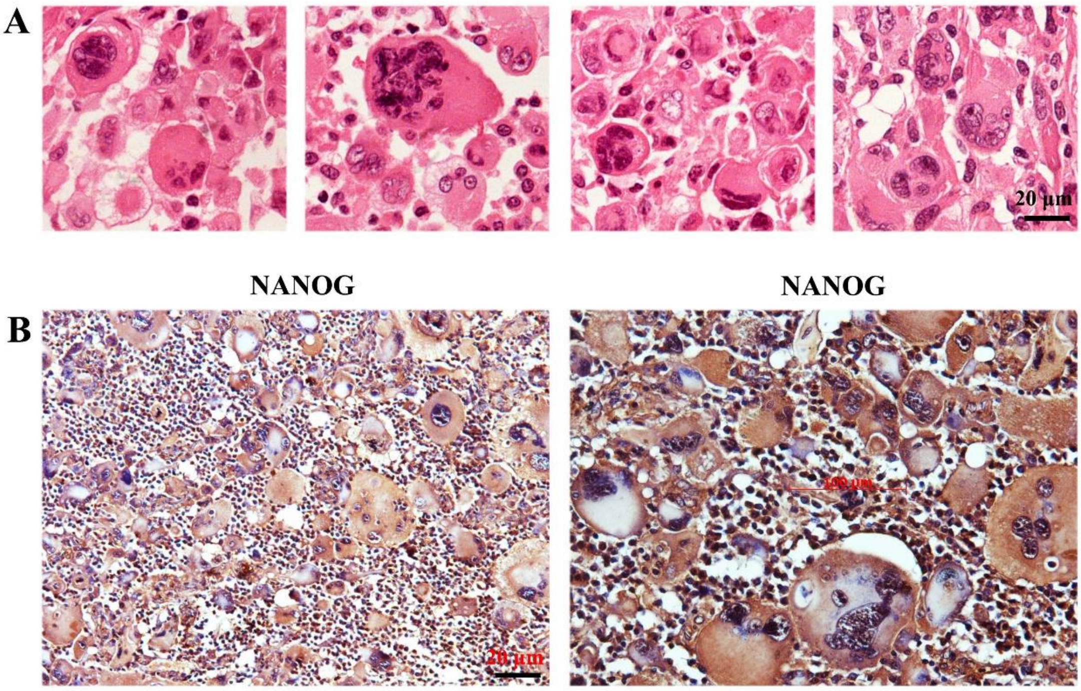

Supplementary Figure S9. Appearance of germ cell-like cells in human glioma tissues. (A) Morphology and marker expression of embryonic/germ cell-like cells in human glioma tissues. (B) HE staining and Immunohistochemistry assays showing the germ cell-like cell and embryo-like structures at different developmental stages.

Supplementary Table S1. Sequencing data of CRIPSR-Cas9 knockout glioma cells.

Mutation	sgRNA target/ mutant target	indels
**A172-Dazl$^{+/-}$**	…ACCATATCTAGCAAAGAAGCTTCTAATCTCA……<br>…ACCATAT----------AAAGAAACTTCTCACCCTCT…	-5 bp
**U251-Dazl$^{+/-}$**	…ACCATATCTAGCAAAGAAGCTTCTAATCTCA……<br>…ACCATATCAA----AAAAAA----TTCTTATCTCC……	-4 bp
**LN229-Dazl$^{+/-}$**	…ACCATATCTAGCAAAGAAGCTTCTAATCTCA……<br>…ACCATA----TAATAAACAACCATTCAATCTA…	-2bp

Supplementary Table S2. Detailed information from the CGGA database regarding mRNA sequencing.

	Sample Number				
	**Total**	**WHO II**	**WHO III**	**WHO IV**	
mRNAseq_325 for figure 3-5.	313 (4 without WHO information)	98	75	136	Data without survival information was deleted. Data with incomplete survival less than three years was deleted.
Combined with mRNAseq_325 and mRNAseq_693 for figure 6. Some data without 1p19q status	898	242	301	352	Data without survival information was deleted. Data without WHO classification was deleted.

Supplementary Table S3. Comparison of gene expression in subtype of gliomas with distinct WHO classification (Unpaired t test with Welch's correction).

	Mean ± SEM						
**Genes**	**WHO II-P (n=232)**	**WHO II-R (n=59) (Vs II-P)**	**WHO III-P (n=194) (Vs II-P)**	**WHO III-R (n=140) (Vs III-P)**	**WHO IV-P (n=225) (Vs III-P)**	**WHOIV-R (n=133) (Vs IV-P)**	**WHO IV-S (n=30) (Vs IV-P)**
**POU5F1**	0.31±0.03	0.34 ± 0.06 ns	0.38 ± 0.06 ns	0.34 ± 0.05 ns	0.63 ± 0.12 *	0.62 ± 0.12 ns	0.33 ± 0.05 *
**ROR2**	0.42± 0.10	0.37± 0.08 ns	0.42 ± 0.07 ns	0.80± 0.27 ns	0.67 ± 0.09 *	0.93 ± 0.24 ns	0.45 ± 0.11 ns
**WNT5A**	3.7 ± 0.26	3.10± 0.43 ns	4.42 ± 0.38 ns	4.541± 0.41 ns	5.32 ± 0.33 ns	5.602± 0.60 ns	6.13 ± 0.70 ns
**GDF15**	2.80± 0.58	6.52± 1.32 *	3.29 ± 0.42 ns	6.68 ± 0.76 ****	17.74± 1.97 ****	25.67± 5.39 ns	6.20 ± 1.35 ****
**ITGB1**	30.89±1.46	44.13±4.73 **	45.39±4.44 **	57.48 ± 4.66 ns	78.87± 4.40 ****	81.79± 5.79 ns	114.7±11.59 **
**CXCR4**	12.19±0.97	13.4± 1.81 ns	12.37±0.92 ns	13.39 ± 1.18 ns	28.87± 1.89 ****	27.4 ± 2.67 ns	45.91±13.69 ****
**ZP3**	3.79± 1.60	6.12± 3.16 ns	5.29 ± 1.34 ns	1.93± 0.53 *	3.12 ± 0.52 ns	7.12 ± 4.48 ns	0.68 ± 0.22 ****
**KLF4**	4.27± 0.35	4.12± 0.48 ns	3.13 ± 0.35 ns	4.21 ± 0.64 ns	4.27 ± 0.27 ns	4.77± 0.38 ns	3.13 ± 0.35 *
**MYC**	19.44±1.00	26.94± 3.38 *	21.11 ± 1.6 ns	24.92 ± 1.65 ns	19.71± 1.39 ns	19.37± 1.49 ns	42.27±6.12 **
**STAT3**	28.61±1.02	32.02± 2.45 ns	30.45±1.52 ns	35.54 ± 1.93 *	41.25± 1.55 ****	44.25± 2.12 ns	52.63±3.44 **
**BMP8B**	1.99± 0.17	2.42± 0.28 ns	2.57 ± 0.23 *	3.63±0.42 *	3.07 ± 0.32 ns	3.55 ± 0.38 ns	3.40 ± 0.46 ns
**ACVR1**	7.27± 0.27	8.19 ± 0.56 ns	7.17 ± 0.37 ns	8.40±0.42 *	8.54 ± 0.40 *	8.50± 0.44 ns	11.89±0.64 ****
**PRDM1**	1.25± 0.07	1.19± 0.15 ns	1.39 ± 0.15 ns	1.41± 0.12 ns	2.25 ± 0.14 ****	2.05± 0.18 ns	2.53 ± 0.27 ns
**IFITM3**	160.1±12.3 9	269 ± 35.16 **	226.5±15.32 ***	268.9±24.04 ns	472.2±30.0 ****	556.1±36.7 ns	245.7±45.03 ****
**SOX17**	2.17± 0.13	4.22 ± 0.54 ****	2.30±0.13 ns	3.63±0.64 *	2.25 ± 0.16 ns	2.59 ± 0.29 ns	1.01 ± 0.10 ****
**DMC1**	0.62± 0.08	0.84± 0.17 ns	1.02± 0.14 **	0.73 ± 0.10 ns	1.07 ± 0.17 ns	1.09 ± 0.14 ns	0.47± 0.08 **
**SYCP3**	0.88± 0.08	0.80± 0.11 ns	1.10± 0.11 ns	0.99± 0.11 ns	1.26 ± 0.31 ns	0.82±0.085 ns	0.26± 0.065 **
**DND1**	1.97± 0.13	2.12± 0.29 ns	2.30± 0.16 ns	2.10± 0.19 ns	1.56 ± 0.15 ***	1.46 ± 0.15 ns	0.64± 0.08 ****

**KIT**	4.53± 0.28	3.37± 0.42<br>*	5.42± 1.74<br>ns	3.122 ± 0.24<br>ns	2.8 ± 0.32<br>ns	3.12 ± 0.69<br>ns	4.672 ± 1.34<br>ns
**NANOS3**	21.5± 2.27	29.74±6.19<br>ns	33.5 ± 3.33<br>**	22.33 ± 2.31<br>**	16.33± 1.78<br>****	17.3 ± 2.04<br>ns	5.79±0.71<br>****
**BMP4**	3.65± 0.20	3.92± 0.40<br>ns	3.50± 0.25<br>ns	3.48 ± 0.26<br>ns	2.52± 0.15<br>**	2.56± 0.22<br>ns	2.59± 0.27<br>ns
**BMP2**	17.13±0.99	15.49± 2.49<br>**	12.29 ± 0.96<br>***	14.6 ± 1.43<br>ns	5.44 ± 0.61<br>****	5.84± 0.89<br>ns	12.48± 2.34<br>**
**DDX4**	0.06± 0.01	0.07± 0.01<br>ns	0.07 ± 0.01<br>ns	0.08 ± 0.01<br>ns	0.09 ± 0.01<br>*	0.05 ± 0.01<br>**	0.05 ± 0.01<br>**
**SOX2**	154.5± 6.23	180.8±14.2<br>ns	170.1 ± 8.50<br>ns	198.2±11.52<br>ns	149 ± 6.87<br>ns	167.6 ±9.80<br>ns	181.2±15.05<br>ns
**NOTCH1**	23.42 ±0.85	22.82± 2.11<br>ns	24.47 ± 1.04<br>ns	25.69 ± 1.49<br>ns	19.43±0.97<br>***	19.49 ±1.25<br>ns	25.86± 3.62<br>ns

Supplementary Table S4. Comparison of gene expression in gliomas with distinct WHO classifications (Unpaired t test with Welch's correction and Mann Whitney test).

	**Mean ± SEM**				
**Genes**	**LGG (n=625)**	**GBM(n=88)**	**sGBM(n=0)**	**LGG&GBM**	**LGG&sGBM**
POU5F1	0.3402 ± 0.02	0.60± 0.08	0.33± 0.05	0.0014**	0.7699 ns
SOX2	171.6 ± 4.59	157.9 ± 5.36	181.2± 15.05	0.0516 ns	0.5442 ns
KLF4	4.092 ± 0.213	4.35± 0.21	3.126 ± 0.35	0.3837 ns	0.0224*
MYC	21.89 ± 0.79	21.34 ± 1.11	42.27 ± 6.12	0.6842 ns	0.0025**
STAT3	31.05 ± 0.78	43.16 ± 1.19	52.63 ± 3.44	<0.0001****	<0.0001****
LIF	1.633 ± 0.20	8.631 ± 1.20	9.81± 6.95	<0.0001****	<0.0001**** (MW test)
NOTCH1	24.2 ± 0.60	19.95 ± 0.76	25.86± 3.624	<0.0001****	0.6539 ns
ITGB1	42.59 ± 1.91	82.65 ± 3.38	114.7± 11.59	<0.0001****	<0.0001****
CXCR4	12.63 ± 0.56	29.68 ± 1.78	45.91± 13.69	<0.0001****	<0.0001**** (MW test)
ROR2	0.50± 0.07	0.74± 0.10	0.45± 0.11	0.0469 *	0.7243 ns
WNT5A	4.05 ± 0.18	5.48± 0.28	6.131± 0.70	<0.0001****	0.0070**
BMP8B	2.58 ± 0.14	3.258 ± 0.23	3.40 ± 0.46	0.0112*	0.0042**** (MW test)
ACVR1	7.58± 0.19	8.79± 0.29	11.89 ± 0.64	0.0004***	<0.0001****
PRDM1	1.32±0.06	2.20 ± 0.10	2.53 ± 0.27	<0.0001****	0.0001***
IFITM3	215.3 ± 9.31	483.5±22.08	245.7± 45.03	<0.0001****	0.5136 ns
GDF15	4.17± 0.33	19.57 ± 2.19	6.201 ± 1.35	<0.0001****	0.1542 ns

ZP3	4.06± 0.79	4.30 ± 1.57	0.68± 0.22	0.8882 ns	<0.0001****
DND1	2.12 ± 0.09	1.46 ± 0.10	0.64± 0.08	<0.0001****	<0.0001****
NANOS3	26.14 ± 1.554	15.85 ± 1.26	5.79 ± 0.71	<0.0001****	<0.0001****
KIT	4.39 ± 0.56	3.06 ± 0.2	4.67 ± 1.34	0.0383*	0.8443 ns
BMP4	3.59 ± 0.13	2.54± 0.12	2.60 ± 0.27	<0.0001****	0.0020**
BMP2	14.89 ± 0.62	6.12 ± 0.51	12.48 ± 2.34	<0.0001****	0.3262 ns
SYCP3	0.96± 0.05	1.03 ± 0.18	0.26± 0.06	0.7364 ns	<0.0001****
DMC1	0.79 ± 0.06	1.03 ± 0.11	0.47 ± 0.09	0.0551 ns	0.0029**
DDX4	0.08 ± 0.004	0.06 ± 0.004	0.05± 0.01	0.0001***	0.0140*
SOX17	2.73± 0.17	2.27 ± 0.14	1.01 ± 0.10	0.0351*	<0.0001****

Supplementary Table S5. Comparison of the median survival of gliomas with distinct gene expression.

	Median overall survival			
**Genes**	**Low (n=485)**	**High (n=485)**	**P**	**P Summary**
POU5F1	1321	824	0.0004	***
SOX2	1054	970	0.2773	ns
KLF4	1079	952	0.238	ns
MYC	1023	1008	0.1466	ns
NOTCH1	842	1197	0.0868	ns
BMP2	506	2304	<0.0001	****
BMP4	745	1321	<0.0001	****
BMP8B	2219	660	<0.0001	****

LIF	2499	484	<0.0001	****
STAT3	2199	609	<0.0001	****
ACVR1	1252	782	<0.0001	****
PRDM1	1933	681	<0.0001	****
IFITM3	2367	540	<0.0001	****
ITGB1	2527	523	<0.0001	****
CXCR4	2199	576	<0.0001	****
WNT5A	1977	681	<0.0001	****
ROR2	2156	623	<0.0001	****
SOX17	863	1122	0.0806	ns
KIT	723	1588	<0.0001	****
NANOS3	825	1244	0.0038	**
DND1	809	1252	0.0044	**
DDX4	788	1188	0.0067	**
SYCP3	921	1068	0.2298	ns
DMC1	1252	834	0.0065	**
ZP3	1134	827	0.0531	ns
GDF15	2630	473	<0.0001	****

Supplementary Table S6. Comparison of gene expression in gliomas with distinct 1p19q codeletion statue (Unpaired t test with Welch's correction).

Genes	Mean ± SEM		P	P	Mean ratio
	Codel n=212	Non-codel n=728		Summary	Codel/Noncode
POU5F1	0.21 ± 0.01	0.55 ± 0.05	<0.0001	****	0.375
SOX2	180.90 ± 7.57	173.80 ± 4.07	0.4119	ns	1.04
KLF4	3.52 ± 0.22	4.723± 0.20	<0.0001	****	0.75
MYC	27.11 ± 1.67	21.25 ± 0.72	0.0014	**	1.28
NOTCH1	27.14 ± 0.99	22.21 ± 0.55	<0.0001	****	1.22
BMP2	26.15 ± 1.32	8.14 ± 0.36	<0.0001	****	3.21
BMP4	4.11 ± 0.21	3.084 ± 0.11	<0.0001	****	1.33
BMP8B	0.78 ± 0.06	3.62 ± 0.16	<0.0001	****	0.22
LIF	0.71 ± 0.17	5.554 ± 0.67	<0.0001	****	0.13
STAT3	26.92 ± 1.00	40.61 ± 0.83	<0.0001	****	0.66

ACVR1	7.55 ± 0.31	8.83 ± 0.19	0.0004	***	0.85
PRDM1	1.10 ± 0.06	1.95 ± 0.07	<0.0001	****	0.56
IFITM3	146.10 ± 7.69	397.20 ± 14.03	<0.0001	****	0.37
ITGB1	34.53 ± 1.86	69.59 ± 2.35	<0.0001	****	0.5
CXCR4	10.73 ± 0.65	23.38 ± 1.06	<0.0001	****	0.46
WNT5A	3.53 ± 0.35	5.20 ± 0.19	<0.0001	****	0.68
ROR2	0.13 ± 0.02	0.76 ± 0.08	<0.0001	****	0.17
DND1	2.35 ± 0.16	1.87 ± 0.08	0.0072	**	1.26
NANOS3	28.27 ± 2.83	22.52 ± 1.23	0.0636	ns	1.26
KIT	5.19 ± 0.30	3.83 ± 0.49	0.019	*	1.36
DDX4	0.07 ± 0.01	0.08 ± 0.004	0.0794	ns	0.86
DMC1	0.56 ± 0.07	1.02 ± 0.07	<0.0001	****	0.55
SYCP3	1.06 ± 0.09	1.06 ± 0.10	0.9977	ns	1
SOX17	2.63 ± 0.15	2.72 ± 0.15	0.6723	ns	0.97
ZP3	8.91 ± 3.47	3.15 ± 0.36	<0.0001	****	2.82
GDF15	2.30 ± 0.28	13.11 ± 1.22	<0.0001	****	0.17

Supplementary Table S7. RNA sequencing cut-off values of the genes in the molecular group.

**Genes**	**Cut off**
LIF	3.8
ITGB1	60
CXCR4	30
ROR2	2.3
WNT5A	7.5
STAT3	59
IFITM3	390
GDF15	16.9
ACVR1	16.4
BMP8B	5
PRDM1	5
POU5F1	2

Supplementary Table S8. Comparison of the median survival of gliomas with distinct classifications.

Classification	Median survival		P value
	High	Low	
Total (MG only)	447 (n=520)	3411 (n=378)	****P<0.0001
WHO IV+MG	344 (n=307)	710 (n=47)	****P<0.0001
WHO III+MG	554 (n=143)	2633 (n=158)	****P<0.0001
WHO II+MG	2219 (n=70)	>5000 (n=172)	****P<0.0001
1p19q-codel+MG	1265 (n=41)	>5000 (n=138)	****P<0.0001
1p19q-noncodel+MG	415(n=463)	2382 (n=186)	****P<0.0001
IDH-WT+MG	387 (n=323)	1196 (n=57)	****P<0.0001
IDH-Mut/1p19q-non+MG	723 (n=160)	2552 (n=136)	****P<0.0001
IDH-Mut/1p19q-cod+MG	>5000 (n=43)	>5000 (n=128)	**P=0.0044

Note: Molecular group (MG)

Supplementary Table S9. Positive ratio of molecular groups in distinct glioma types.

LIF, STAT3, PRDM1, IFITM3, ACVR1, CXCR4, WNT5A, ROR2, ITGB1, POU5F1, GDF15 or BMP8B		
**types**	**Positive ratio**	**Compare**
WHO II-Total (n=242)	29.33%	&WHO III-T: ****P<0.0001<br>& WHO IV-T: ****P<0.0001

WHO II-Primary (n=202)	24.71%	&WHO II-R: **P=0.0011<br>&WHO III-P: **P=0.0015<br>& WHO IV-P: ****P<0.0001<br>& WHO IV-S: ****P<0.0001
WHO II-Recurrent (n=40)	52.50%	
WHO III-Total (n=301)	41.19%	& WHO IV-T: ****P<0.0001<br>& WHO IV-T: ****P<0.0001
WHO III-Primary (n=181)	40.88%	& WHO III-R: **P=0.0033<br>& WHO IV-P: ****P<0.0001<br>& WHO IV-S: ****P<0.0001
WHO III-Recurrent (n=120)	58.3%	
WHO IV-Total (n=354)	87.28%	
WHO IV-Primary (n=206)	83.98%	& WHO IV-R: P=0.1818 ns<br>& WHO IV-S: ****P<0.0001
WHO IV-Recurrent (n=119)	89.91%	
WHO IV-Secondary (n=29)	100%	

Supplementary Table S10. Comparison of the median survival of gliomas with the WHO classification.

	**WHO classification**			
	**WHO II**	**WHO III**	**WHO IV**	**P value**
Median survival	>5000 (n=242)	1109 (n=301)	349 (n=352)	****P<0.0001

Supplementary Table S11. Comparison of the median survival of gliomas with the WHO classification.

**IDH-1p19q classification**

	IDH-Mut/1p19q-cod	IDH-Mut/1p19q-non	IDH-WT	P value
Median survival	>5000 (n=171)	1258 (n=296)	420 (n=380)	****P<0.0001

Supplementary Table S12. Expression ratio of proteins related to embryonic/germ cells in glioma tissues.

	POU5F1/SOX2/NANOG/NANOS3/DDX4/DAZL	
	**Sigle positive**	**All positive**
WHO IV (n=20)	80%	50%
WHO III (n=8)	62.5%	37.5%
WHO II (n=10)	40%	20%

Images of the original blots

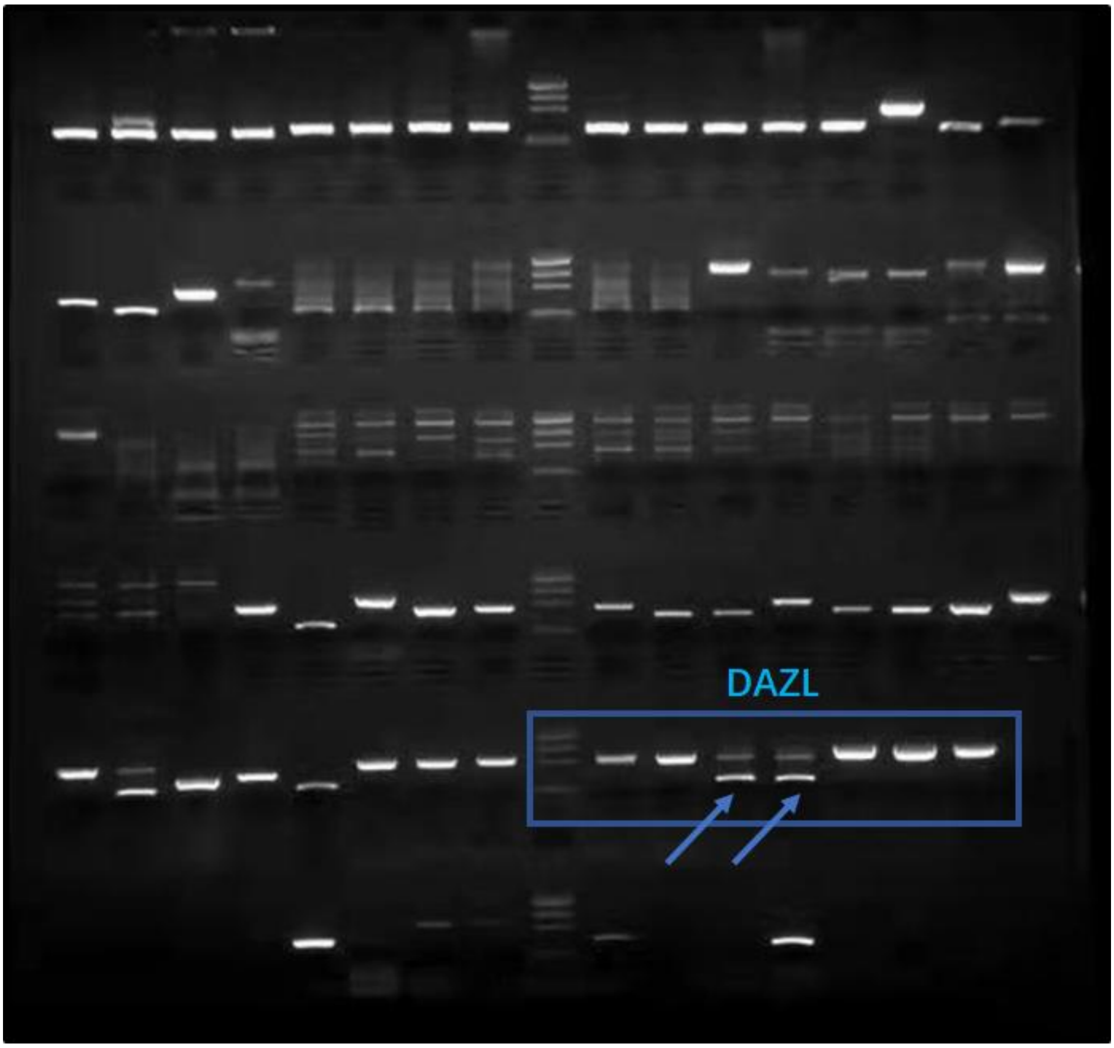

Full PCR of gels from Supplementary Figure S1. PCR showed the DAZL with heterozygote (arrow).
